# Characterizing donation behavior from psychophysiological indices of narrative experience

**DOI:** 10.3389/fnins.2015.00301

**Published:** 2015-08-31

**Authors:** Kelly A. Correa, Bradly T. Stone, Maja Stikic, Robin R. Johnson, Chris Berka

**Affiliations:** Advanced Brain Monitoring, Inc.Carlsbad, CA, USA

**Keywords:** EEG, narrative, donation, prosocial behavior, HRV, affect

## Abstract

Research on narrative persuasion has yet to investigate whether this process influences behavior. The current study explored whether: (1) a narrative could persuade participants to donate to a charity, a prosocial, behavioral decision; (2) psychophysiological metrics can delineate the differences between donation/non-donation behaviors; and (3) donation behavior can be correlated with measures of psychophysiology, self-reported reactions to the narrative, and intrinsic characteristics. Participants (*n* = 49) completed personality/disposition questionnaires, viewed one of two versions of a narrative while EEG and ECG were recorded, completed a questionnaire regarding their reactions to the narrative, and were given an opportunity to donate to a charity related to the themes of the narrative. Results showed that: (1) 34.7% of participants donated; (2) psychophysiological metrics successfully delineated between donation behaviors and the effects of narrative version; and (3) psychophysiology and reactions to the narrative were better able to explain the variance (88 and 65%, respectively) in the amount donated than all 3 metrics combined as well as any metric alone. These findings demonstrate the promise of narrative persuasion for influencing prosocial, behavioral decisions. Our results also illustrate the utility of the previously stated metrics for understanding and possibly even manipulating behaviors resulting from narrative persuasion.

## Introduction

Storytelling has existed throughout human history and across cultures as a means of conveying information. It is a powerful tool with the potential to influence understanding, emotions, beliefs, and even alter behavior. Due to this potential, methods for improving narrative messaging could be useful in multiple domains such as marketing, consumer neuroscience, entertainment, jury decision-making, prosocial/health messaging, public safety, public service announcements, etc. Much of the existing literature on the persuasive power of narratives has focused on how transportation during a narrative affects this process (Green and Brock, 2000; McFerran et al., 2010; Banerjee and Greene, 2012; Van Laer et al., 2012; Murphy et al., 2013). Transportation has been conceptualized as the level of understanding and attention paid to the narrative, the ability to imagine the narrative world, and the ability to feel strong emotions, especially empathy, toward the characters and events within the narrative (Van Laer et al., 2012). This strong connection to a story is theorized to explain how changes in beliefs occur after reading or listening to a narrative (Green and Brock, 2000).

Green and Brock (2000) tested the Transportation Theory to see if, in fact, transportation can increase the persuasive power of a narrative. They created a self-report measure of transportation and found that highly transported individuals are more likely to adopt the values of a narrative into their own personal beliefs and are less likely to find faults in a narrative. This study and measure of transportation sparked further research on the involvement of this concept in the narrative persuasion process. Van Laer et al. (2012) performed a meta-analysis on the resulting literature across multiple disciplines. They found that increases in transportation are mediated based on whether the narrative contains characters with whom the audience can identify and empathize, a plot that is conducive to the audience's imagination, and a plot that is believable. Transportation also depends on audience characteristics such as their familiarity with the topic, how attentive they are to the narrative, and how transportable they are. Furthermore, Van Laer et al. (2012) found that transportation can increase emotions, thoughts, beliefs, attitudes, and intentions that are consistent with those of the narrative, while also reducing critical thoughts. These findings not only demonstrate the importance of transportation in narrative persuasion, but also highlight the significance of intrinsic audience characteristics, narrative content, and most importantly, the potential for behavior change via narrative messaging due to its effects on cognition and affect.

While transportation does seem to play an important role in narrative persuasion overall, these effects and processes may differ with various direct applications. Consumer psychology and marketing research focuses directly on the persuasive effects of narratives. Narrative advertisements have, so far, proven effective by increasing self-brand connections, which lead to greater likelihoods of purchasing as well as more positive attitudes toward the brand (Escalas, 2004). The use of narratives was also found to be more favorable than ads employing a non-narrative format, even when undesirable details are mentioned (Adaval and Wyer, 1998). However, these effects are not standard across all consumers. Mattila (2000) investigated the impact of a consumer's prior familiarity with a product and found that narrative ads are effective in changing consumer's emotions when they are unfamiliar with the topic, but that ad format has no effect on expert consumers' feelings (Mattila, 2000). Instructions on what to focus on in an ad may aid this process as well. The combination of intrinsic motivation and instruction to immerse oneself in a narrative ad increases transportation, which in turn leads to increased likelihood of purchasing (McFerran et al., 2010). These studies provide further evidence for the utility of narratives and transportation in the persuasion process as well as stress the importance of evaluating intrinsic qualities of the audience.

The utility of narrative persuasion has also caught the attention of researchers in the fields of prosocial/public health messaging who hope to utilize this tool with more of a humanitarian focus on the power of persuasion. These fields seek to increase understanding, awareness, and healthy behaviors and could benefit from narrative persuasion. When compared to traditional health messages (statistics, risk factors, symptoms, etc.), narratives were found to be more effective in aiding these efforts by increasing knowledge and positive attitudes in regards to health issues (Murphy et al., 2013). Transportation seems to, again, be a key factor in these outcomes because the more an individual is transported, the more health-related knowledge they will gain from a narrative (Murphy et al., 2013). Additionally, individuals who are highly transported while reading anti-drug narratives are more likely to have greater cognitive and affective responses, which lead to a greater likelihood of reporting negative views on drugs (Banerjee and Greene, 2012). While research on narrative persuasion in prosocial/health messaging has shown that it can impact the decision making process, the exact benefits of using narratives to deliver this type of information, as well as the mechanisms behind how this persuasion occurs, are still unclear (Winterbottom et al., 2008). Due to the potential for positive outcomes for many individuals, further exploration of narrative persuasion in prosocial/health messaging is needed.

One key aspect necessary for advancing existing knowledge on the utility of narrative persuasion in prosocial/health messaging is the evaluation of the persuasive power of narratives for driving positive, prosocial/health-related behaviors. The majority of literature on narrative persuasion has focused on change related to beliefs and attitudes, yet little has been investigated on the effects that these changes may have on behavioral outcomes. In order to augment understanding on this issue, we conducted an exploratory study that revolved around the following hypotheses: (1) a narrative could persuade audience members to donate to a charity, a prosocial, behavioral decision; (2) psychophysiological metrics obtained from the audience during the presentation of a narrative can delineate the differences between donation behaviors; and (3) donation behavior can be correlated with measures of psychophysiology, reactions to the narrative, and intrinsic characteristics of audience members.

Previous research on narrative persuasion has mainly focused on self-report measures to evaluate how cognitive and affective responses affect the reader or listener. However, recent literature on prosocial behaviors has begun focusing on psychophysiological measures to investigate these processes. Studies utilizing fMRI have revealed that systems related to rewards and social attachment are active during prosocial behaviors (Moll et al., 2006; Harbaugh et al., 2007). While these findings provide information about the processes that occur as one performs a prosocial behavior, they do not delineate what types of psychophysiological patterns lead up to such behaviors. The current study evaluated the underlying processes of narrative persuasion by exploring whether a variety of psychophysiological metrics are related to these processes and any resultant prosocial behaviors. We used the conceptualization of transportation described by Van Laer et al. (2012) and evaluatad psychophysiological metrics associated with its key components: attention, imagination, and emotions (particularly looking at empathy). Attention was assessed through validated EEG (electroencephalography) measures of engagement (Berka et al., 2007; Johnson et al., 2011) and workload (Berka et al., 2005, 2007). The role of imagination was examined through the slower range of the Alpha frequency band (8–10 Hz) in the occipital and parietal regions, as these areas have been negatively correlated with visual (O1 and O2) and kinesthetic (P3 and P4) mental imagery (Cremades and Pease, 2007).

For the analysis of strong emotions, including empathy, we looked at EEG power spectral densities (PSDs), wavelets, and measures of heart rate and heart rate variability. Heart rate (HR) was evaluated due to its relationship with changes in emotion and corresponding levels of arousal (Peira et al., 2013) during the presentation of affective films/narratives (Carvalho et al., 2012; Fernández et al., 2012). We analyzed heart rate variability (HRV) because it has been widely associated with emotion regulation (Segerstrom and Nes, 2007; Denson et al., 2011; Koval et al., 2013) and has a strong relationship with the autonomic nervous system in regards to an adaptive stress response (Berntson and Cacioppo, 2004; Tan et al., 2010). Using PSDs and wavelets, an EEG-based classifier was applied to further distinguish neurophysiological signatures of positive and negative affect (Stikic et al., 2014). Lastly, empathy was evaluated through the EEG measure of mu suppression, which is related to increases in response to social and interactive stimuli (Oberman et al., 2007), self-reported levels of empathy (Woodruff et al., 2011), and empathy for someone else's pain regardless of their similarity to one's self (Perry et al., 2010).

Additional EEG correlates were examined because of their implications for narrative persuasion through transportation. Midline theta activity was investigated due to its associations with encoding into, and retrieval from, long term memory (Klimesch et al., 2010), visual stimuli matching long term episodic memories (Gruber et al., 2008; Zion-Golumbic et al., 2010), sustained or concentrated attention (Inanaga, 1998; Aftanas and Golocheikine, 2001; Missonnier et al., 2006; Doppelmayr et al., 2008; Kao et al., 2013), visual working memory (Gevins et al., 1997; Jensen and Tesche, 2002; Onton et al., 2005), positive emotions (Aftanas and Golocheikine, 2001; Sammler et al., 2007), and decreased levels of anxiety (Inanaga, 1998). These processes not only relate to the attention and strong emotion aspects of transportation, but may also have implications for how memory processes affect narrative persuasion. The role of prefrontal gamma was also investigated. The few existing neuroimaging studies on narratives indicate that the prefrontal cortex may play an important role in the underlying processes behind narrative persuasion (Fletcher et al., 1995; Maguire et al., 1999; Mar, 2004). Activation in this region was found to be associated with the attribution of mental states to characters (Fletcher et al., 1995) as well as narrative comprehension (Maguire et al., 1999). We chose to investigate the role of gamma band activity in this region due to its relationships with perception (Castelhano et al., 2013) and attention (Fries et al., 2001; Womelsdorf et al., 2006) in general. Specifically, in the prefrontal cortex, gamma band activity is associated with visual attention (Gregoriou et al., 2009), working memory (Gou et al., 2011; Roux et al., 2012), language abilities (Gou et al., 2011), and visual working memory (Polanía et al., 2012). All of these processes potentially play key roles in narrative comprehension and retention as well as relate to the attention component of transportation.

In addition to psychophysiological metrics, the current study examined self-report measures of intrinsic audience characteristics as well as audience reactions to the narrative characters. Since these factors have been known to affect transportation (Mattila, 2000; McFerran et al., 2010; Van Laer et al., 2012), we sought to elucidate their roles in behavioral outcomes with respect to the psychophysiological metrics. We assessed the effects of intrinsic audience characteristics through subjective measures of empathy, anxiety, and depression due to recent findings that showed that personality characteristics, mediated by physiology (oxytocin), impact prosocial behaviors such as donating to a charitable organization (Barraza and Zak, 2009; Barraza et al., 2011; Hoge et al., 2012). Broader personality factors were also explored due to their relationships with prosocial behaviors (Blanca et al., 2007; Steele et al., 2008). Since association with characters can influence transportation (Van Laer et al., 2012), as well as the adaptation of attitudes held by narrative characters (de Graaf et al., 2012), we investigated whether increased identification, likeability, worthiness, and sympathy for a protagonist or supporting character, as well as opposite ratings for an antagonist, would relate to prosocial, behavioral decisions.

## Materials and methods

### Participants

Healthy participants (*n* = 54) between the ages of 18 and 70 years old were recruited through web-based advertisements that targeted residents of San Diego County, advertisements posted in college newspapers, and flyers distributed to student groups at the local colleges. The exclusion criteria for participation consisted of the following factors that can alter EEG or impair the participant's ability to complete the study: any known sleep, neurological, pulmonary, psychiatric, behavioral, attention, or eating disorders; regular use of medication other than over-the-counter medication; head injury within the last 5 years; an older head injury with current symptoms; illegal drug use; history of substance abuse; smoking more than 10 cigarettes per day; drinking more than 5 alcoholic beverages or 4 caffeinated beverages per day; use of amphetamines; high blood pressure; heart disease; diabetes; history of stroke; pregnancy; breastfeeding; and untreated or untreatable vision or auditory issues. Additionally, participants were required to refrain from alcoholic beverages 24 h prior to their study, caffeinated beverages 12 h prior to their study, and nicotine 1 h prior to their study. Furthermore, participants were required to refrain from using the aforementioned substances for the duration of their study visits. Five participants were not included in the analysis due to non-compliance with the study protocol. This resulted in a final sample size of 49 participants that were 42.9% male, 91.8% Caucasian, and 10.2% Hispanic or Latino with a mean age of 40.7 years old (range = 19–66 years old).

All participants received compensation ($20/h) for taking part in the current study. The use of human participants was approved by the Chesapeake Institutional Review Board prior to participant recruitment.

### Materials/equipment

#### Psychophysiology

EEG and electrocardiography (ECG) were acquired throughout the three testing sessions, using the B-Alert® X24 wireless sensor headset (Advanced Brain Monitoring, Inc., Carlsbad, CA). This system had 19 referential EEG channels located according to the International 10–20 system at Fp1, Fp2, Fz, F3, F4, F7, F8, T3, T4, T5, T6, Cz, C3, C4, Pz, P3, P4, O1, and O2. There was also an additional referential EEG channel located at POz (required to run the B-Alert engagement and workload classifications) as well as an auxiliary channel for ECG. Linked reference electrodes were located behind each ear on the mastoid bone. ECG electrodes were placed on the right clavicle and the lower left rib. Data were sampled at 256 Hz with a high band pass at 0.1 Hz and a low band pass, fifth order filter, at 100 Hz obtained digitally with Sigma-Delta A/D converters. Artifacts are automatically detected and decontaminated in the time-domain EEG signal that include 3, 5, or 7 data point spikes with amplitudes greater than 40 mV (caused by tapping or bumping of the sensors), amplifier saturation, and excursions that occur during the onset or recovery of saturations (Berka et al., 2004; Johnson et al., 2011). Data were transmitted wirelessly via Bluetooth to a host computer up to 10 m from the sensor headset. Data acquisition software then stored the psychophysiological data on the host computer. The proprietary acquisition software also included artifact decontamination algorithms for eye blink, muscle movement, and environmental/electrical interference such as spikes and saturations.

#### Narrative

The researchers collaborated with a professional storyteller, Kendall Haven, in order to construct a contemporary narrative that focused on the themes of fairness and justice. A videographer recorded the professional storyteller as he performed a live version of said narrative. The video displayed the storyteller from the shoulders up to remove the possibly confounding variables of hand gesturing and arm movements. Contact the corresponding author for further details, or access, in regards to these videos.

The narrative centered around a young, Hispanic woman who was putting herself through school by working as a night janitor in an office building. She was late to work one night as she hurried past Freight, a man with a mental disability, stopping only to hear him tell a quick joke. She then dashed upstairs to clean a conference room where a meeting was being held by Ramon, a wealthy and politically powerful man. As Mary bent down to pick up a trash can, Ramon commented on and patted her rear. When Mary rushed out of the room, Ramon demanded that she bring coffee refills to which Mary replied “I just clean. Get it yourself.” Later that night, Ramon confronted Mary on her walk to the bus stop. She decided to stand up for herself, again, which enraged Ramon. He retaliated and assaulted Mary by slamming a sizeable rock into her face. Freight came to Mary's aid and attacked Ramon until the police arrived and intervened.

The narrative was developed in 11 segments; three of the segments had alternate versions. The depictions of the protagonist, Mary, and the antagonist, Ramon, each had two alternate versions that were designed to evoke varying degrees of empathy from the audience. One was a positive portrayal created so that the audience would empathize with the protagonist and antagonist more. The other was a negative portrayal created so that the audience would empathize with the protagonist and antagonist less. The resolution of the narrative had two distinct versions that varied in levels of injustice (least just vs. most just). These alternate segment versions resulted in eight possible versions of the narrative. Two narrative versions were ultimately used in this study: (1) the least just resolution with the character descriptions that audience was anticipated to empathize with the least and (2) the most just version with the character descriptions that the audience was anticipated to empathize with the most. It should be noted, however, that none of the resolutions were meant to be perfectly ideal; even the most just version did not result in significant punishment for the crime committed. A more detailed description of each segment and which narrative version they belonged to can be found in Table 1.

**Table 1:** **Narrative segments and corresponding versions**.

	**Segment description**	**Narrative version**
1	Introduction: Narrative takes place in San Francisco on 10/14/2008	Both
2A	Protagonist Introduction: less empathetic toward Mary	Least just
2B	Protagonist Introduction: more empathetic toward Mary	Most just
3	Provides additional background on Mary and her current activities	Both
4	Supporting character introduction	Both
5	Supporting character interacts with the protagonist	Both
6	Ramon holds a meeting as Mary cleans the room	Both
7A	Antagonist Introduction: less empathetic toward Ramon	Least just
7B	Antagonist Introduction: more empathetic toward Ramon	Most just
8	Ramon pats and comments on Mary's rear; Mary stands up for herself	Both
9	Ramon confronts Mary outside and she stands up for herself again	Both
10	Ramon attacks Mary; Freight attacks Ramon to save Mary	Both
11A	Ramon serves 3 weeks in jail; Both Mary's and Freight's needs are recognized and met	Most just
11B	Ramon is revered as a hero for supposedly saving Mary from Freight; Mary is deported to Mexico after her wounds are treated; Freight is sent to prison for the alleged assault and teased by the prison guards nightly	Least just

The least just version described the protagonist, Mary, as a hard-working and unassertive woman whose arduous past was unfortunate, undeserved, and unfair. This version of Mary was designed to elicit less empathy, due to her inability to assert herself. Ramon, the antagonist, was depicted as manipulative, arrogant, and egotistical. This depiction also aimed at evoking less empathy from the audience. The resolution of this narrative consisted of Ramon testifying that Mary was actually a prostitute who had attacked him. As a result of this lie, Mary unfairly received a severe punishment from the court and continued to be victimized by her circumstances. In contrast, Ramon remained wealthy, in power, and was rewarded for his actions.

Alternatively, the most just version elicited more empathy toward Mary by depicting her as a strong, independent woman, capable of working the system in her favor in order to succeed in life. This version also engendered more empathy toward Ramon by depicting him as a hard-worker and proud of his family's heritage. The resolution of the most just version was designed to be neutral; justice was served, but Mary was not the clear winner and Ramon's punishment was light in respect to his crime.

#### Post-narrative questionnaire

The Post-Narrative Questionnaire was a self-report questionnaire designed to evaluate audience reactions to the narrative, how much of it they paid attention to and remembered, and how it affected their donation behaviors. There were 33 questions in total and the response options included free response, multiple choice, and a 7 point Likert scale. The Likert scale utilized anchors at every point on the scale, ranging from 1 being a very negative trait to 7 being a very positive trait. The questionnaire consisted of three sections: (1) questions 1–18 asked about the participant's reactions to the narrative and its characters (e.g., How likeable is Mary?; How worthy is Ramon of receiving good things/outcomes?; How sympathetic are you to Freight?; How did the ending of the story make you feel?); (2) questions 19–22 pertained to the donation; and (3) question 23–33 served as the memory portion of the questionnaire and contained one multiple choice question for each of the 11 narrative segments.

Participants were informed of the donation portion of the questionnaire during orientation and were told that all donations were completely voluntary, but that if they did choose to donate, the donation amount would be directly deducted from their compensation for participating in the study. Question 19 allowed participants to choose one of three charities to donate to (if inclined to do so): Women's Resource Center (an organization that arranges for shelter and support for battered women), Harvest of Hope (a Migrant farm worker charity), or ARC (a charity that serves those with intellectual and developmental disabilities). Question 20 presented a donation range of $0–40. The limited range was based on the minimum amount of time (2 h) that the participant was expected to be involved in the study. For further details, or to obtain a copy of this questionnaire, contact the corresponding author.

#### Neurocognitve assessment

The Alertness and Memory Profiler (AMP)™ is a customized, computer-based software tool developed to time the presentation of each stimulus in each neuropsychological task as well as record user responses to every stimuli presented. The AMP output files contained the simultaneously acquired EEG and ECG signals with markers denoting when each stimulus was presented, when each stimulus response occurred, and a description of each stimulus response. While there are 11 neurocognitive tasks in total, only the 3 vigilance tasks were utilized for the current study. These 3 tasks are described below in the same order they were presented to participants. All tasks provided a train-to-criterion practice portion, reducing the potential learning effects that could occur otherwise.

The three choice active vigilance task (3CVT) is a 20 min long task that requires participants to discriminate one target (70% occurrence) from two non-target (30% occurrence) geometric shapes. Each stimulus is presented for a duration of 200 ms. The inter-stimulus interval is variable and was changed for each quartile of the task: 1–3 s for the 1st quartile, 1–6 s for the 2nd and 3rd quartiles, and 1–10 s for the last quartile. Participants were instructed to respond as quickly as possible to each stimulus by selecting the left arrow for target stimuli and the right arrow for non-target stimuli.

The other two vigilance tasks are passive vigilance tasks that last 5 min each. The visual psycho-vigilance task (VPVT) repeatedly presents a 10 cm circular target image for a duration of 200 ms. The target image is presented every 2 s in the center of the computer monitor. The auditory psycho-vigilance task (APVT), which participants completed with their eyes closed, consists of an auditory tone that is played every 2 s. For both passive vigilance tasks, participants were asked to tap the spacebar when the stimulus (red circle or auditory tone) was presented.

### Procedures

Participants came in for three sessions: orientation, neurocognitive assessment, and narrative. All three sessions were completed within a 2 month time span. Potential participants had to first complete a brief telephone screener in order to be eligible for the orientation session. On the day of their orientation session, they consented for the study and completed a longer, more thorough eligibility screener in order to confirm their eligibility for the study. If they were eligible for the study, they then completed the Beck Depression Inventory (BDI) (Beck et al., 1996), the State Trait Anxiety Inventory (STAI) (Spielberger et al., 1983), the International Personality Item Pool Representation of the NEO Personality Inventory-Revised (IPIP NEO PI-R) (McCrae and Costa, 2010), and the Interpersonal Reactivity Index (IRI) (Davis, 1980) in order to measure intrinsic characteristics that may be related to transportation. Each participant scheduled their next session, the neurocognitive assessment, within 2 weeks after completing their orientation.

Neurocognitive assessments began between 8:00 and 8:30 a.m. These were individual sessions that lasted around 3 h. Participants were first set up with the B-Alert® X24 headset and then completed the Alertness and Memory Profiler (AMP)™. The assessment included the three previously described tasks, in the order described above, as well as eight additional tasks that were not utilized for the current analyses.

All narrative sessions were conducted at 7:30 and 9:30 a.m. and were counterbalanced by narrative version (least just vs. most just). Each session consisted of three participants and lasted 2–3 h long. All participants were set up with the B-Alert® X24 headset upon arrival. Next, they watched the narrative video as a group (*n* = 3/group). Note that, out of the final *n* = 49 participants used for analyses, *n* = 23 participants watched the least just version, while *n* = 26 watched the most just version. A video projector was utilized in order to display a 5′ by 6.5′ projection of the videos against a wall in a 17.5′ by 7.5′ room. Participants were seated in a row of three chairs that were placed five feet from the wall upon which the videos were projected. Once the video finished, the participants were seated in separate rooms to complete the Post-Narrative Questionnaire.

### Data analysis

Mu suppression was calculated using log ratios of the power spectral densities (PSDs) across sites C3, Cz, and C4 from 8 to 13 Hz bins of the participant's experimental and baseline tasks (Oberman et al., 2007; Singh et al., 2011). The APVT, from the neurocognitive assessment, was used as the baseline task for Mu suppression calculations. Midline theta was calculated by summarizing PSDs from 3 to 7 Hz bins across sites Fz, Cz, Pz, and POz. Prefrontal Gamma was measured by combining the PSDs from 25 to 40 Hz bins of sites Fp1 and Fp2. The engagement and workload metrics were calculated with reference to the 3CVT, APVT, and VPVT tasks from the neurocognitive assessment (Berka et al., 2005, 2007; Johnson et al., 2011). An affective state classifier was used, which indicated the probabilities of a positive (values greater than 0.5) affective state being experienced and negative (values less than 0.5) affective state being experienced (Stikic et al., 2014). The current study chose to utilize the generalized version of the quadratic discriminant function analysis from Stikic et al. (2014) based on their findings. Lastly, HRV was calculated using power spectral computations across 5 min blocks of HR to derive the ratio (LF:HF) of low frequency (LF) HRV to high frequency (HF) HRV. HRV LF:HF ratios were calculated in order to determine whether the sympathetic nervous system, or the parasympathetic nervous system, was dominant during each narrative segment (Camm et al., 1996). Low frequency HRV was calculated as the sum of the power spectrum from 0.04 to 0.15 Hz, whereas, high frequency HRV was the sum of the power spectrum from 0.15 to 0.4 Hz. A summary of the metrics and related constructs can be found in Table 2.

**Table 2:** **All metrics and corresponding constructs**.

**Metrics**	**Construct**
**PSYCHOPHYSIOLOGICAL**	
Heart rate variability	Emotion regulation
Affective state classifier	Emotional valence
Engagement classifier	Engagement
Midline theta	Attention, memory encoding and retrieval, positive emotions, and relaxation
Heart rate	Emotions and arousal
Mu suppression	Empathy
Prefrontal gamma	Perception, attention, memory, and narrative comprehension
Workload classification	Workload
Left occipital alpha slow suppression	Visual imagery
Right occipital alpha slow suppression	Visual imagery
Left parietal alpha slow suppression	Kinesthetic imagery
Right parietal alpha slow suppression	Kinesthetic imagery
**PERSONALITY**	
NEO personality inventory-revised	Personality
Interpersonal reactivity index	Empathy
Beck depression inventory	Depression
State-trait anxiety inventory	Anxiety
**NARRATIVE**	
Post-story questionnaire	Reactions to the narrative

## Results

### Efficacy of narrative manipulations

In order to ensure that the participants understood, and paid attention to, the details of the narrative, we evaluated their performance on the memory portion of the Post-Narrative Questionnaire. All of the final *n* = 49 participants had a minimum accuracy score of at least 73% and a maximum score of 100% on the memory portion of the questionnaire (*M* = 87.38, *SD* = 10.78). A Mann-Whitney *U*-test was performed to verify whether the degree of empathy evoked from the audience for the protagonist and antagonist was successfully manipulated by narrative version as well as to verify the efficacy of the manipulation of justice in the narrative resolution by narrative version. There were no statistically significant differences between narrative versions on how likeable, sympathetic, and worthy participants rated the protagonist and antagonist to be. However, there were significant differences in the ratings of satisfaction with the resolution of the narrative. Ratings of resolution satisfaction from those who viewed the most just version (mean rank = 33.78) were significantly greater than for those who viewed the least just version (mean rank = 14.41), *U* = 519.5, *z* = 4.942, *p* < 0.0005.

### Donation behavior and self-reported ratings

In total, 34.7% of the participants chose to donate; 21.7% of those who watched the least just version donated and 46.2% of those who watched the most just version donated. Those participants who did choose to donate, donated an average amount of $6.74 (range = $1.50–10.00), with 52.9% donating to ARC, 35.3% to Women's Resource Center, 5.9% to Harvest of Hope, and one participant choosing to divide up their donation across all three charities. In order to further investigate differences in donation behavior, a second Mann-Whitney *U*-test was performed to see if there was a difference between those who donated and those who did not donate on how likeable, sympathetic, and worthy they rated the characters as well as how satisfied they were with the narrative resolution. There were no significant differences between those who donated and those who did not donate on how satisfied they were with the narrative resolution or how likeable, sympathetic, and worthy they rated the protagonist and antagonist. There were also no significant differences between donation behaviors and how sympathetic and worthy Freight, the supporting character, was rated. However, Freight's likeability ratings did show a difference between donation behaviors. Freight's likeability ratings were significantly higher for those who donated (mean rank = 31.44) than for those who did not donate (mean rank = 20.69), *U* = 381.5, z = 2.726, *p* < 0.01.

### Overall psychophysiological differences between narrative versions and donation behaviors

A 2 × 2 ANOVA was conducted to determine whether there were statistically significant differences in the psychophysiological metrics (refer to Table 2) between the narrative versions (most just and least just) and donation behaviors (donated and did not donate). No significant differences were found for the engagement classifier values, midline theta, heart rate, mu suppression, prefrontal gamma, workload classifier values, left and right occipital alpha suppression, or left and right parietal alpha suppression. The metrics that were found to have significant effects are reported below.

#### Heart rate variability

**Figure 1:**
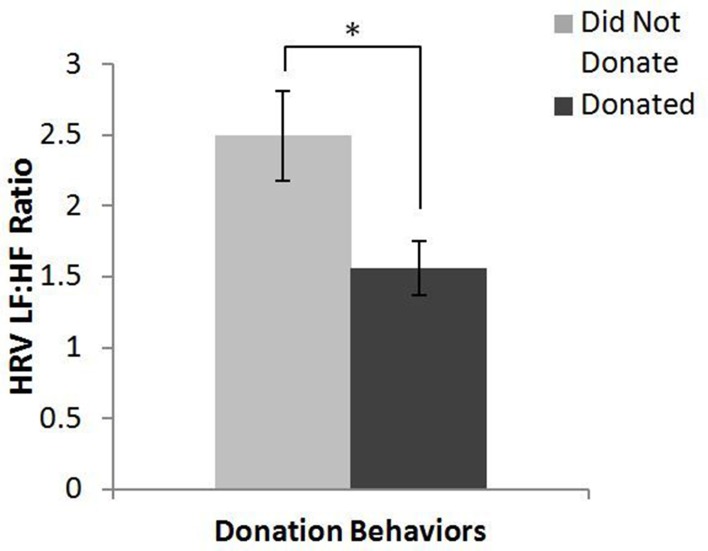
**Main effect of donation behavior on HRV LF:HF ratio**. ^*^p < 0.05.

The main effect of donation behavior, which is presented in Figure 1, showed that those who did not donate (*M* = 2.5, *SD* = 1.8) had statistically significantly greater HRV LF:HF ratios during the narrative than those who donated (*M* = 1.56, *SD* = 0.79), *F*_(1, 45)_ = 5.149, *p* < 0.05.

#### Affective state classifier

**Figure 2:**
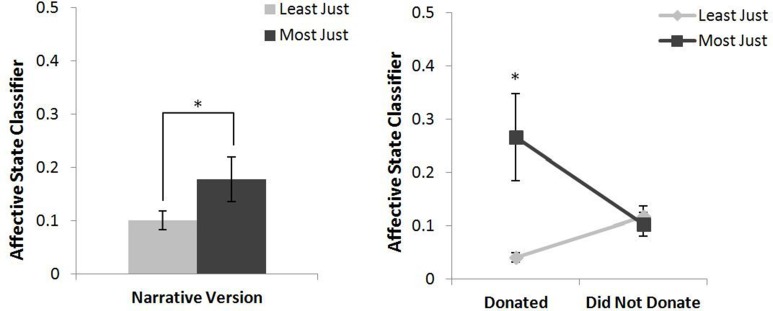
**Main effect of narrative version on negative affective state (left)**. Negative affective state classification between donation behaviors by narrative version (**right**). Smaller values indicate greater negative affect. ^*^p < 0.05.

The main effect of narrative version showed a statistically less negative affective response overall for those who viewed the most just version (*M* = 1.96, *SD* = 2.34) than for those who viewed the least just version (*M* = 1.11, *SD* = 0.93), *F*_(1, 45)_ = 4.455, *p* < 0.05. The results also showed a significant interaction between narrative version and donation behavior for the affective state classifier, *F*_(1, 45)_ = 5.703, *p* < 0.05. *Post-hoc* comparisons using *t*-Tests with a Bonferroni adjustment showed a statistically less negative affective response for those who donated and watched the most just version (*M* = 0.27, *SD* = 0.08) than for those who donated and watched the least just version (*M* = 0.04, *SD* = 0.02), *p* < 0.05; no such differences occurred for those who did not donate. These data are presented in Figure 2.

### Segmented psychophysiological differences between narrative versions and donation behaviors

Seven additional 2 × 2 ANOVA's were ran in order to evaluate the differences in the psychophysiological metrics (refer to Table 2) during specific narrative segments of interest between the narrative versions (most just and least just) and donation behaviors (donated and did not donate). ANOVA results for HRV LF:HF ratio data were only available for segments 4–11 due to the fact that at least 5 min of data is necessary to calculate HRV (Camm et al., 1996). Segments 2, 3, 4, 5, and 7 were analyzed because they introduced the characters and provided additional background about the characters. Segments 9, 10, and 11 were chosen in order to evaluate the impact of the conflict and resolution scenes. As in the overall ANOVA, the results showed no significant differences in the engagement classifier values, midline theta, heart rate, mu suppression, prefrontal gamma, workload classifier values, left and right occipital alpha suppression, or left and right parietal alpha suppression. Additionally, no significant main effects or interactions for any of the psychophysiological metrics were found for segment 3, which provided additional background for the protagonist, or for segment 7, which introduces the antagonist. However, HRV LF:HF ratios and the affective state classifier were, once again, found to have significant effects.

#### Heart rate variability

The ANOVA results for segment 4, which introduces Freight, the supporting character, showed a significant main effect of donation behavior in terms of HRV LF:HF ratios. Those who did not donate (*M* = 2.29, *SD* = 1.66) had significantly higher HRV LF:HF ratios in segment 4 of the narrative than those who did donate (*M* = 1.37, *SD* = 0.69), *F*_(1, 45)_ = 5.204, *p* < 0.05. Segment 5, which provides further information about Freight by displaying his jovial personality, also showed significantly greater HRV LF:HF ratios for those who did not donate (*M* = 2.46, *SD* = 1.81) than for those who did donate (*M* = 1.63, *SD* = 0.78), *F*_(1, 45)_ = 4.080, *p* < 0.05. These findings demonstrate the utility of HRV LF:HF ratios for differentiating between donation behaviors during the introduction of the supporting character who later becomes the hero of the narrative.

#### Affective state classifier

While the ANOVA results for the affective state classifier during segment 2, which pertains to the protagonist's initial introduction, revealed no main effects for donation behavior or narrative version, the interaction results were able to provide additional information on the differences between donation behaviors. The interaction showed a significantly more negative affective response for those who donated and viewed the least just version of segment 2 (*M* = 0.04, *SD* = 0.03) in comparison to those who donated and viewed the most just version of the segment (*M* = 0.29, *SD* = 0.33), *F*_(1, 45)_ = 7.333, *p* < 0.05. No significant differences between the versions were found for those who did not donate.

The affective state classifier was also able to differentiate between donation behaviors, as well as narrative version, during the supporting character's interaction with the protagonist. The main effect of narrative version for segment 5 showed that those who viewed the least just version of the narrative had a significantly more negative affective response during segment 5 (*M* = 0.08, *SD* = 0.11) than those who viewed the most just version (*M* = 0.22, *SD* = 0.28), *F*_(1, 45)_ = 6.413, *p* < 0.05. The ANOVA for segment 5 also revealed a significant interaction, such that those who donated and viewed the least just version (*M* = 0.03, *SD* = 0.02) had a significantly more negative affective response during segment 5 than those who donated and viewed the most just version (*M* = 0.33, *SD* = 0.37), *F*_(1, 45)_ = 4.549, *p* < 0.05; no such differences occurred between versions for those who did not donate.

Affective state classifier values during the conflict and resolution segments revealed additional differences between the narrative versions as well as a difference between donation behaviors, depending on narrative version, during the resolution. The ANOVA results from the scenes depicting the conflict between the protagonist and antagonist (segments 9 and 10), both showed a main effect of narrative version for the affective state classifier. Those who viewed the least just version (*M* = 0.08, *SD* = 0.07) had a significantly more negative affective response during segment 9 than those who watched the most just version (*M* = 0.20, *SD* = 0.26), *F*_(1, 45)_ = 5.283, *p* < 0.05. The same main effect of narrative version was found for segment 10 as well; those who viewed the least just version (*M* = 0.08, *SD* = 0.09) had a significantly more negative affective response than those who watched the most just version (*M* = 0.17, *SD* = 0.24), *F*_(1, 45)_ = 4.333, *p* < 0.05. The ANOVA for segment 11, the resolution of the narrative, showed a main effect of narrative version as well as an interaction between donation behavior and narrative version for the affective state classifier. Those who viewed the least just version of the resolution (*M* = 0.10, *SD* = 0.11) had a significantly more negative affective response during the resolution segment than those who viewed the most just version of the resolution (*M* = 0.16, *SD* = 0.17), *F*_(1, 45)_ = 4.448, *p* < 0.05. Additionally, those who donated and viewed the least just version of the resolution (*M* = 0.03, *SD* = 0.02) had a significantly more negative affective response than those who donated and viewed the most just version of the resolution (*M* = 0.23, *SD* = 0.22), *F*_(1, 45)_ = 5.882, *p* < 0.05, but no significant differences in affective state classifier values occurred between narrative versions for those who did not donate.

### Personality differences between donation behaviors

A One-Way ANOVA was conducted to test for differences across the personality metrics between those who donated and those who did not donate. The results showed that those who donated had significantly higher IRI scores (*M* = 98.18, *SD* = 10.64) than those who did not donate (*M* = 86.97, *SD* = 14.92), *F*_(1, 47)_ = 7.524, *p* < 0.01. Three IPIP NEO PI-R subfactors differentiated between the two groups as well. Those who donated had higher levels of morality (*M* = 64.29, *SD* = 27.28) than those who did not donate (*M* = 46.03, *SD* = 27.93), *F*_(1, 46)_ = 4.77, *p* < 0.05. Levels of sympathy were higher for those who donated (*M* = 64.88, *SD* = 26.22) than for those who did not donate (*M* = 45.32, *SD* = 26.27), *F*_(1, 46)_ = 6.096, *p* < 0.05. Lastly, those who donated had significantly higher levels of dutifulness than (*M* = 69.1, *SD* = 17.16) those who did not donate (*M* = 53.94, *SD* = 27.71), *F*_(1, 46)_ = 4.163, *p* < 0.05.

### Characterizing donation behavior

In addition to exploring differences between individuals who donated, and not, further analyses were employed to assess whether these variables could explain donation behaviors. Eight forward step-wise linear multiple regression analyses were ran to (1) test if physiology alone, personality alone, narrative reactions alone, or the combination of all three correlated to the amount of money participants donated, and (2) to see which of these explained the greatest amount of the variance in donation behaviors. Due to the large amount of variables and potential for multicollinearity, principal component analyses (PCAs) were conducted on the predictor variables for each of the regressions. Since PCAs are sensitive to the scaling of variables, all variables were z-scored prior to these analyses. The resulting components from these PCAs were then used as predictor variables for the step-wise regressions. In order to fully explore which variables can best explain donation behaviors, two sets of PCAs and corresponding regressions were conducted. The first set included all participants while the second set only included those who donated. Refer to Table 2 for a list of metrics and to the Supplemental Tables for weights of all variables in each component entered into the step-wise linear multiple regressions.

#### Personality

Principal component analyses were performed on all of the z-scored personality measures. Step-wise multiple regressions were then conducted using the resultant components as the independent variables and the amount donated as the dependent variable. The first set of analyses, which included all participants, showed that the first two PCA components were statistically significant in explaining the variance in the amount of money a participant donates, *R*^2^ = 0.39, adj. *R*^2^ = 0.36, *F*_(2, 39)_ = 12.543, *p* < 0.001. The next set of analyses, which only included those who donated, revealed that the first two components significantly explained the variance in the amount of money a participant donated, *R*^2^ = 0.49, adj. *R*^2^ = 0.41, *F*_(2, 12)_ = 5.808, *p* < 0.05.

#### Psychophysiology

Two principal component analyses were performed on all of the z-scored psychophysiological measures. Corresponding step-wise multiple regressions were then conducted using the resulting components to see how much of the variance in amount donated could be explained by these metrics alone. The segmented psychophysiological measures were entered in due to the ANOVAs' findings on individual segments that revealed significant relationships with donation behaviors. The analyses including all participants showed that the first 19 components from the corresponding PCA could significantly explain the variance in donation behavior, *R*^2^ = 0.93, adj. *R*^2^ = 0.88, *F*_(19, 29)_ = 20.367, *p* < 0.001. The set of analyses that only included those who donated showed that only the first component was able to statistically significantly explain the variance in donation amounts, *R*^2^ = 0.40, adj. *R*^2^ = 0.36, *F*_(1, 15)_ = 10.114, *p* < 0.05.

#### Narrative reactions

A third set of PCAs were performed using the z-scored narrative reaction measures. The components found by these analyses were then entered as predictor variables into two step-wise multiple regressions. These regressions were used to investigate how much of the variance in amount donated these measures could explain without including the personality or psychophysiological measures. The analyses which included all participants showed that the first two components significantly explained the variance in donations, *R*^2^ = 0.31, adj. *R*^2^ = 0.28, *F*_(2, 43)_ = 9.584, *p* < 0.001. For the analyses that only included those who donated, the first three components significantly explained the variance in donation amount, *R*^2^ = 0.72, adj. *R*^2^ = 0.65, *F*_(3, 13)_ = 10.975, *p* < 0.001.

#### Personality, psychophysiology, and narrative reaction

The personality, segmented psychophysiology, and narrative reaction measures were entered into two PCAs and corresponding step-wise multiple regression analyses. The set of analyses including all participants found that the first six components were significant explanatory variables of donation behavior, *R*^2^ = 0.50, adj. *R*^2^ = 0.41, *F*_(6, 34)_ = 5.568, *p* < 0.001. When only those who donated were included into the analyses, only the first component significantly explained the variance in the amount of money a participant donated, *R*^2^ = 0.40, adj. *R*^2^ = 0.35, *F*_(1, 13)_ = 8.558, *p* < 0.05.

**Figure 3:**
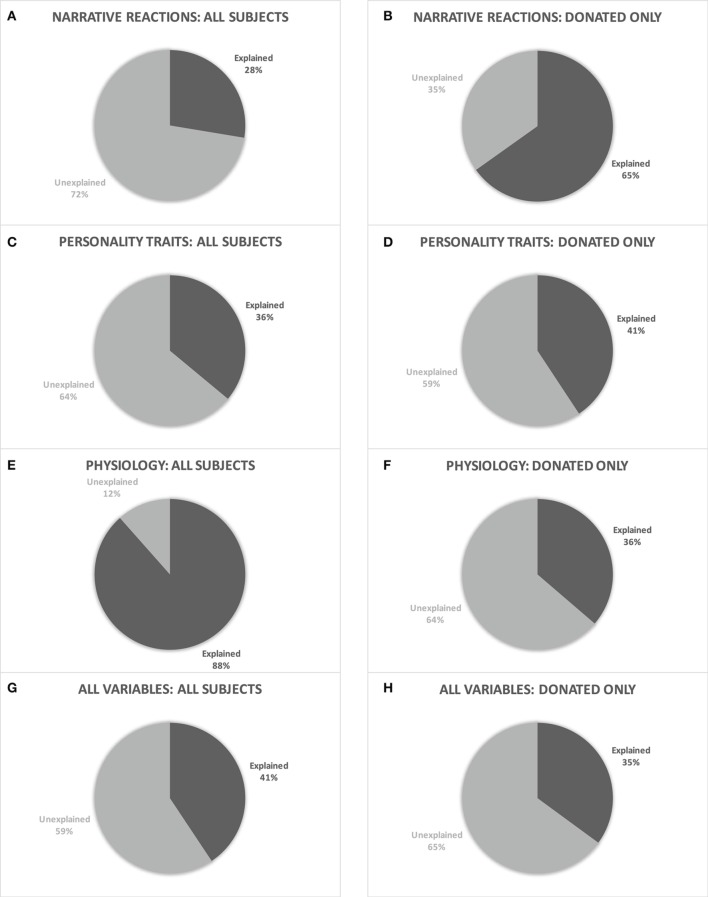
Pie chart representations of the explanatory power of narrative reactions for (A) all subjects and (B) subjects who donated. Personality Traits for (C) all subjects and (D) subjects who donated. Physiology for (E) all subjects and (F) subjects who donated, and all metrics combined for (G) all subjects and (H) subjects who donated.

See Figure 3 for a visual representation of the regression results, depicting how much variance in the amount donated could be explained by each individual analysis.

## Discussion

Narrative persuasion holds great potential as a means for increasing the efficacy of prosocial/health messaging (Winterbottom et al., 2008; Banerjee and Greene, 2012; Murphy et al., 2013). In order to further evaluate its utility in this domain, the current study sought to investigate whether narrative persuasion could influence behavioral outcomes, distinguish which measures of psychophysiology were integral to this process, and explain the variance in the amount of money participants donated based on psychophysiological metrics, measures of personality and disposition, and reactions to the narratives.

Some participants did, in fact, make a prosocial, behavioral decision to donate to a charity after viewing the narrative (34.7% across both versions). This allowed us to investigate differences in narrative persuasion between those who chose to donate and those who did not. Since de Graaf et al. (2012) and Van Laer et al. (2012) showed that identifying with narrative characters influences the audience to adapt attitudes and beliefs similar to those of a character that they identify with, we explored whether this adaptation of attitudes and beliefs could influence behaviors. We expected that those who identified with the characters, and consequently donated, would rate the protagonist more positively, the supporting character more positively, and the antagonist more negatively than those who did not identify with the characters or donate. However, the results did not fully support this notion. Ratings of how sympathetic, likeable, and worthy the antagonist and protagonist were, as well as satisfaction with the narrative resolution, did not differ between donation behaviors. Although there were also no differences on ratings of how sympathetic and worthy Freight, the supporting character, was, we did find that those who donated perceived Freight to be more likeable than those who did not donate. While Freight was initially evaluated as a supporting character, it is possible that he was perceived as the hero of the story and was consequently more galvanizing of a character than previously expected. Due to character identification's influence on attitudes and beliefs, it is possible that those who found Freight to be more likeable were more prone to emulate his willingness to help others in need by making a charitable donation.

The findings regarding narrative versions validated the manipulations of fairness and justice by showing that audiences did perceive a difference between the narrative resolutions. The resolution of the most just version received greater satisfaction ratings than the least just version. However, our manipulations of the portrayals of the protagonist and antagonist failed to yield any differing perceptions across narrative versions. According to self-reported, post-narrative perceptions, the two versions of the segments which introduce the antagonist, as well as the two versions of the segments which introduce the protagonist, can be interpreted similarly across narrative versions. In contrast, the least just and most just resolution segments can be viewed as distinct from one another. The results based on the psychophysiological measures both supported and provided additional details on these differences between versions.

The ANOVA results on overall psychophysiological measures further clarified the different ways in which the narrative versions affected the audience members as well as the differences between those who donated and those who did not. In comparison to those who donated, those who did not donate showed an increase in overall HRV LF:HF ratios regardless of which narrative version they watched. Increased HRV LF:HF ratios reflect increased sympathetic nervous system activation, an indicator of the fight or flight response, and have been related to poor decision making (Fenton-O'Creevy et al., 2012; Laborde and Raab, 2013). Although HRV was initially investigated due to its relationship with strong emotions (Segerstrom and Nes, 2007; Denson et al., 2011; Koval et al., 2013), an aspect of transportation, in addition to its relationship with a stress response (Berntson and Cacioppo, 2004; Tan et al., 2010), it is possible that this increase in stress affected their decision making processes during the narrative as well as during the opportunity to donate. Alternatively, HRV is also positively correlated with self-regulatory effort, which can be depleted by previous exertions of such effort (Segerstrom and Nes, 2007). If the increased overall HRV in those that did not donate was a reflection of their levels of self-regulatory efforts during the narrative (e.g., maintaining focus and attention), their subsequent deficit in self-regulatory effort may have made it more difficult to choose to donate and help others instead of receiving the entirety of their compensation for themselves. There was also a significant main effect of narrative version. While audiences from both versions experienced negative affective states, the affective state of those who watched the least just version was significantly more negative than those who viewed the most just version. This result, combined with the lower satisfaction ratings for the least just version, indicates that our manipulation was successful in making the least just version more negative overall than the most just version.

The interaction between donation behavior and narrative version provides additional insight into the ways in which those who choose to donate differ from those who do not. Those who donated and viewed the least just version experienced a significantly more negative affective state than those who donated and watched the most just version. In contrast, affective states did not significantly differ across versions for those who did not donate. This interaction result suggests that those who donate are more responsive to the overall emotional valence of a narrative. Furthermore, this finding, based on an objective and quantifiable measure of affective valence, supports previous, self-report-based literature on narrative persuasion, which suggests that feeling strong emotions for the characters and events within a narrative is a key aspect of transportation during a narrative (Green and Brock, 2000; Van Laer et al., 2012). Consequently, there may be intrinsic differences between those who are likely to donate and those who are not.

The findings from the ANOVA's on the character introduction, climax, and resolution segments provide further support for these differences between donation behaviors as well as clarify which segments of the narrative best display this effect. Similar to the findings on self-reported narrative perceptions, no significant effects were found for the segment which introduces the antagonist. However, the results related to the protagonist's introduction did show significant differences between the versions that the self-report measures were not able to detect. The ANOVA for the protagonist's introduction showed that those who donated experienced a significantly more negative affective state when viewing the less empathetic depiction of the protagonist in the least just version than when viewing the more empathetic portrayal of the protagonist in the most just version. No significant differences between versions existed for those who did not donate. This finding provides additional support for the conceptualization of transportation presented by Green and Brock (2000), which includes feeling strong emotions toward narrative characters as a key aspect of transportation. Furthermore, it suggests that being differentially affected by more or less empathetic protagonist introductions is a key indicator of later donation behavior.

The supporting character's introduction and interaction with the protagonist also provide insight into the effects of narrative version and differences between donation behaviors. These two segments yielded greater sympathetic activation (HRV LF:HF ratios) in those who did not donate as opposed to those who did, which implies that they may have found these segments to be more stressful. The increased stress could have moderated the ability of those who did not donate to identify and empathize with the supporting character and consequently affected their decision to donate. These segments may also have affected donation behaviors by eliciting increased levels of self-regulatory effort and creating subsequent deficits in such resources in those who did not donate. The interaction between these two characters influenced the affective states of the audience as well. Participants who viewed the most just version experienced less of a negative affective state when the supporting character interacted with and told a joke to the protagonist as opposed to those who viewed the least just version. More specifically, the interaction revealed that this relationship only existed for those who donated. It's possible that the least just version, which depicted the protagonist as less empathetic than the most just version, affected the audience's perception of her, especially for those who donated, and therefore made this interaction less enjoyable.

The climax and resolution segments yielded affective state differences between narrative versions and donation behaviors as well. Participants who viewed the least just version of the narrative experienced significantly more negative affective states during the climax segments than those who viewed the most just version. This finding needs further exploration in order to facilitate possible interpretations. A similar main effect of narrative version was found for the resolution as well. The least just version of the resolution produced significantly more negative affective states than the most just resolution. The interaction results for this segment clarified the difference by showing that, similar to the protagonist and supporting character interaction segment, the significant difference in affective states between versions only existed for those who donated. This result further demonstrates the efficacy of our manipulation of the resolution segments, provides additional support for the theory that feeling strong emotions for narrative events is an important aspect of transportation, and implies that the ability to be differentially affected by more or less just resolutions may also be an indicator of post-narrative decisions to donate. Overall, the ANOVA findings demonstrate the importance of HRV LF:HF ratios and affective reactions during the introduction of the positive characters, the climax scenes, and the resolution of the narrative. These findings also demonstrate that those who make a post-narrative donation are more affected by changes in valence than those who do not donate and provide insight into how these reactions might affect later perceptions and decisions.

In addition to supporting the idea that intrinsic differences affect the efficacy of narrative persuasion (Mattila, 2000; McFerran et al., 2010; Van Laer et al., 2012), dispositional and personality differences between donation behaviors also support the notion of intrinsic differences between those who donate and those who do not. Participants who donated had significantly higher levels of empathy, morality, sympathy, and dutifulness than those who chose not to donate. Previous studies have reported similar findings in relation to donation behavior and being concerned for others, such that those who report a willingness to donate are more empathetic than those who do not (Blanca et al., 2007), empathy affects one's decision of how much money to donate (Barraza and Zak, 2009; Dickert et al., 2011), and sympathy mediates attitudes toward donations (Massi Lindsey and Ah Yun, 2005). The higher levels of morality and dutifulness (the need to fulfill moral obligations) for those who donated expand upon existing literature by suggesting that having a concern for others may not be the only pertinent characteristic that motivates prosocial behaviors, but that a sense of and need to do the right thing coupled with this concern could be key to explaining what propels one toward action.

The results from the regressions demonstrate that reactions to the narrative, psychophysiology, personality, and dispositional characteristics are all, individually, able to explain the variances in donation amount and that the combination of these variables can do so as well. However, psychophysiology and reactions to the narrative were individually able to explain the greatest amount of variance when assessing all participants and only those who donated, respectively. Psychophysiology explained more of the variance in donation amounts than any of the other regressions that included all of the participants. The variables most frequently found to be of the top five most highly weighted in the PCA component analysis, which were added into the psychophysiology regression, were: HRV LF:HF ratios, affective state classifier values, engagement classifier values, midline theta, and prefrontal gamma, across various segments of the narrative. This provides further support for attention and strong emotions as key components within the conceptualization of narrative transportation described by Green and Brock (2000). Furthermore, this finding expands upon existing narrative persuasion literature by showing that not only can narratives influence prosocial behaviors, but that psychophysiological states during the narrative can be used to explain such behaviors.

In contrast, psychophysiology no longer explained the greatest amount of variance when only those who donated were included in the analyses. In this case, reactions to the narrative explained the most variance in donation amounts. Variables related to the participants' perceptions of Freight, their perceptions of Mary, their satisfaction with the resolution of the narrative, and their accuracy on the memory portion of the Post-Narrative Questionnaire were in the top five most highly weighted variables of at least two of the three PCA components entered into this analysis. On the other hand, perceptions of the antagonist did not contribute to the PCA components as much as the other variables, with ratings of Ramon's likeability only being in the top five most highly weighted variables of the second component entered into the step-wise regression. The inclusion of memory accuracy as a highly weighted variable in the PCA components for this regression provides additional support for the attention component in Green and Brock's (2000) conceptualization of transportation, as attention is integral to retaining and recalling information. These findings support and expand upon Van Laer et al.'s (2012) results which showed that narratives containing characters with whom the audience can identify and empathize can increase transportation. The regression results expand upon Van Laer et al.'s (2012) findings by specifying which types of characters are most important for transportation as well as suggesting that satisfaction with the resolution of the narrative may be another key aspect of narrative transportation. It is possible that the audience's percpetions of the hero and the protagonist are more integral to the transportation process than that of the antagonist.

While the regression results including all participants were not similar to those of the analyses which only included participants who donated, the differing results may provide additional insight into the effects of transportation on prosocial behaviors. These differences may have been due to the fact that the majority of participants did not choose to donate. Because of this, it is possible that findings from analyses including all participants may not be solely explaining variances in donation amounts, but rather, differences in donation behaviors (donated or not) as well. Future research should explore how psychophysiology and reactions to a narrative differentially affect both the decision to donate and the amount one donates. While these findings, in conjunction with the ANOVA results, show promise for the use of psychophysiological metrics in evaluating narrative persuasion, replication of the current study is needed. It is possible that the small sample sizes in the regressions that only included those who donated could overfit certain components of the models presented herein. Therefore, we stress the need for a replication of the current study, especially with larger sample sizes, for further validation of these results.

Future studies investigating the effects of narrative persuasion on prosocial, behavioral decisions should address other limitations as well. The current study did not employ the transportation scale to measure how transported participants were during the narrative. The inclusion of this measure could provide further insight into the cognitive, affective, and physiological processes associated with narrative persuasion. In addition, it would be beneficial for future studies to investigate previous donation behaviors of participants in order to determine how past behaviors affect the efficacy of a narrative in persuading one to make a prosocial, behavioral decision. While the current study was able to expand Green and Brock's (2000) conceptualization of transportation to include psychophysiological metrics (HRV LF:HF ratios, the affective state classifier, the engagement classifier, midline theta, and prefrontal gamma) of attention paid to the narrative and strong emotions toward characters and events within the narrative, further research is needed to investigate whether one's ability to imagine the narrative world can be assessed through psychophysiology as well. Such studies should also investigate the associations between parasympathetically mediated HRV, emotion regulation, and narrative persuasion as these relationships may offer additional insights into the role of HRV in narrative persuasion (Appelhans and Luecken, 2006). Moreover, the utility of a movie format for the narrative, instead of the traditional storytelling format used here, needs to be explored. It's possible that the lack of visible actors was the reason why the current study did not find any significant effects for mu suppression. Previous studies on the relationship between mu suppression and empathy have utilized videos in which the actors are moving and interacting with one another (Oberman et al., 2007; Singh et al., 2011), while the interaction of the characters in the current study's narrative had to be imagined by the audience. It is possible that a traditional movie format for a narrative would produce greater mu suppression than the storytelling format used in this study.

## Conclusions

These findings expand upon the previous, self-report-based literature in regards to narrative persuasion and transportation as well as provide a basis for future research to evaluate the role of psychophysiological correlates during narrative persuasion. The current study was able to demonstrate the importance of utilizing psychophysiological measures to understand narrative persuasion, evaluate how psychophysiological measures during key portions of a narrative were related to autonomously motivated, post-narrative behaviors, as well as explain variances in such behaviors based on intrinsic characteristics of the audience members, reactions to the narrative, and psychophysiology during the narrative. These behaviors were autonomously motivated because participants were informed of the voluntary nature of this decision and reminded several times that they do not have to donate to a charity. Replication of this study, as well as additional research, is needed to determine whether or not the manipulation of these metrics can produce increased rates of prosocial, behavioral decisions, and whether any additional psychophysiological correlates could provide further insight into this process.

## Author note

The views, opinions, and/or findings contained in this article are those of the authors and should not be interpreted as representing the official views or policies, either expressed or implied, of the Defense Advanced Research Projects Agency or the Department of Defense.

## Funding

The work presented herein was conducted under a subcontract to Boeing (Customer Contract # CF-12_DARPA; Subcontract Contract # 608687) and was supported by the Defense Advanced Research Projects Agency through the Narrative Networks program.

### Conflict of interest statement

Authors Robin R. Johnson and Chris Berka are share holders in Advanced Brain Monitoring, which may benefit financially from the publication of these data. The other authors declare that the research was conducted in the absence of any commercial or financial relationships that could be construed as a potential conflict of interest.
