# Supplementary material for: Characterizing donation behavior from psychophysiological indices of narrative experience
Source: Front Neurosci. 2015 Aug 31;9:301. doi: 10.3389/fnins.2015.00301 (PMC4553387; doi:10.3389/fnins.2015.00301)
Supplement: Supplementary file 1 [file DataSheet1.PDF]

Contents

Variable Legend 3

ANOVAs - Overall 4

- Personality Metrics 4
- Physiological Metrics - Donation 7
- Physiological Metrics - Narrative Version 8
- Physiological Metrics - Donation*Narrative Version 9
- Physiological Metrics - Donation*Narrative Version (Post-Hoc) 10

ANOVAs - Narrative Segments 11

- Segment 1 11
- Segment 2 13
- Segment 3 15
- Segment 4 18
- Segment 5 20
- Segment 6 23
- Segment 7 25
- Segment 8 27
- Segment 9 30
- Segment 10 33
- Segment 11 35

Principal Component Weights 39

Narrative Reactions 39

Personality 39

Psychophysiology 40

All Metrics Combined 44

Forward Step-wise Linear Multiple Regression Analyses 50

Narrative Reactions 50

Personality 50

Psychophysiology 50

All Metrics Combined 51

Variable Legend

Note: The tables found throughout this document contain abbreviated labels for simplicity in using SPSS. However, when interpreting the meaning of the analyses herein, the authors have provided the following table which details the meanings of the corresponding abbreviations. Within the analyses, a number follows directly after each abbreviated label. This number corresponds to the segment from which the data was analyzed.

Abbreviated Label	Label Meaning
HRV	Heart Rate Variability
EMGQ	Affective State Classifier
ENG	Engagement Classifier
FMT	Midline Theta
HR	Heart Rate
Mu	Mu Suppression
PFG	Prefrontal Gamma
WL	Workload Classification
O1_AS	Left Occipital Alpha Slow Suppression
O2_AS	Right Occipital Alpha Slow Suppression
P3_AS	Left Parietal Alpha Slow Suppression
P4_AS	Right Parietal Alpha Slow Suppression

ANOVAs - Overall

Personality Metrics

Variable	Donation	x̄	σ	df	MS	F	p
**Personality Metrics - Donation**							
BDI	N	4.04	4.83	1	14.80	0.49	0.49
	Y	5.27	6.65				
STAI State	N	31.54	9.66	1	112.33	0.01	0.904
	Y	31.94	12.11				
STAI Trait	N	32.92	9.87	1	111.276	0.19	0.664
	Y	34.38	11.67				
IRI	N	86.97	14.92	1	1394.56	7.52	0.009
	Y	98.18	10.64				
Extraversion	N	61.84	28.40	1	149.55	0.20	0.653
	Y	65.53	24.33				
Friendliness	N	63.94	23.66	1	380.64	0.69	0.412
	Y	69.82	23.38				
Gregariousness	N	60.58	29.22	1	604.37	0.76	0.387
	Y	68.00	25.95				
Assertiveness	N	56.94	28.24	1	66.71	0.10	0.759
	Y	54.47	22.75				
Activity	N	55.84	25.44	1	823.82	1.21	0.278
	Y	47.18	27.37				
Excitement Seeking	N	57.58	31.96	1	27.43	0.03	0.859
	Y	56.00	23.30				
Cheerfulness	N	53.90	30.65	1	885.19	1.10	0.301
	Y	62.88	23.74				
Agreeableness	N	55.23	27.09	1	2396.50	3.39	0.072
	Y	70.00	25.67				
Trust	N	53.19	29.10	1	795.55	1.01	0.321
	Y	61.71	26.26				
Morality	N	46.03	27.93	1	3661.50	4.77	0.034
	Y	64.29	27.28				
Altruism	N	61.90	26.21	1	1366.33	2.29	0.137

	Y	73.06	20.68				
Cooperation	N	58.36	26.34	1	631.88	0.84	0.366
	Y	65.94	29.59				
Modesty	N	52.97	20.24	1	37.58	0.08	0.774
	Y	51.12	23.01				
Sympathy	N	45.32	26.27	1	4200.46	6.10	0.017
	Y	64.88	26.22				
Conscientiousness	N	56.58	26.86	1	203.17	0.28	0.599
	Y	60.88	26.97				
Self-Efficacy	N	58.87	28.37	1	49.77	0.06	0.807
	Y	61.00	29.37				
Orderliness	N	50.36	25.47	1	87.41	0.12	0.73
	Y	53.18	29.55				
Dutifulness	N	53.94	27.71	1	2511.11	4.16	0.047
	Y	69.06	17.16				
Achievement Striving	N	54.71	24.81	1	484.54	0.77	0.385
	Y	61.35	25.66				
Self-Discipline	N	63.03	24.84	1	782.83	0.99	0.325
	Y	54.59	33.42				
Cautiousness	N	49.10	30.13	1	616.17	0.75	0.392
	Y	56.59	25.95				
Neuroticism	N	29.07	25.74	1	41.61	0.05	0.822
	Y	27.12	33.03				
Anxiety	N	30.23	22.48	1	28.03	0.04	0.835
	Y	31.82	29.88				
Anger	N	34.68	24.52	1	234.20	0.30	0.585
	Y	30.06	33.18				
Depression	N	31.52	26.72	1	90.38	0.11	0.742
	Y	28.65	32.07				
Self-Consciousness	N	31.58	23.40	1	0.65	0.00	0.974
	Y	31.82	27.00				
Immoderation	N	40.94	23.95	1	386.80	0.63	0.431
	Y	35.00	26.17				
Vulnerability	N	35.03	26.34	1	117.22	0.15	0.697
	Y	31.77	29.93				
Openness to Experience	N	49.48	28.07	1	1056.65	1.45	0.235

	Y	59.29	24.86				
Imagination	N	46.45	22.92	1	257.46	0.44	0.509
	Y	51.29	26.22				
Altruistic	N	51.00	27.63	1	1474.36	1.93	0.172
	Y	62.59	27.68				
Emotionality	N	44.71	28.04	1	180.53	0.22	0.642
	Y	48.77	29.85				
Adventurousness	N	58.10	28.17	1	390.27	0.51	0.478
	Y	64.06	26.47				
Intellect	N	46.45	28.56	1	706.00	0.82	0.369
	Y	54.47	30.60				
Liberalism	N	54.32	26.89	1	108.67	0.14	0.709
	Y	51.18	29.19				

Donation: Y = Donated, N = Did not Donate; $\bar{x}$ = Mean; σ = Standard Deviation; df = degrees of freedom; MS = Mean Square; F = Mean Square Factor/Mean Square residual; p = Probability of Significance; $\alpha = 0.05$

Physiological Metrics - Donation

Physiological Metrics – Donation						
Variable	Donation	$\bar{x}$	σ	df	F	p
HRV	N	2.495849	1.790648	1	5.15	0.03
	Y	1.560981	0.79458			
EMGQ	N	0.111236	0.084967	1	0.74	0.39
	Y	0.199769	0.256838			
ENG	N	0.400857	0.153468	1	0.03	0.86
	Y	0.422527	0.145775			
FMT	N	3.309019	0.216645	1	1.37	0.25
	Y	3.255971	0.232852			
HR	N	67.61503	9.802595	1	0.34	0.56
	Y	70.56016	10.1096			
Mu	N	0.233932	0.242945	1	0.20	0.66
	Y	0.176258	0.347339			
PFG	N	2.635711	0.239447	1	0.01	0.92
	Y	2.585302	0.271978			
WL	N	0.576789	0.127322	1	0.52	0.48
	Y	0.530528	0.147522			
O1_AS_Su	N	0.471597	0.350541	1	0.06	0.81
	Y	0.391332	0.491645			
O2_AS_SU	N	0.550678	0.374467	1	0.16	0.69
	Y	0.421125	0.54284			
P3_AS_Su	N	0.317834	0.315177	1	0.04	0.85
	Y	0.243608	0.560604			
P4_AS_Su	N	0.347381	0.327359	1	0.23	0.63
	Y	0.241225	0.54028			

Donation: Y = Donated, N = Did not Donate; $\bar{x}$ = Mean; σ = Standard Deviation; df = degrees of freedom; F = Mean Square Factor/Mean Square residual; p = Probability of Significance; α = 0.05

Physiological Metrics - Narrative Version

Physiological Metrics - Narrative Version						
Variable	Narrative Version	x̄	σ	df	F	p
HRV	114	25.36651	19.49892	1	0.264	0.61
	221	22.5774	15.44556			
EMGQ	114	1.107568	0.928182	1	4.455	0.04
	221	1.962989	2.344656			
ENG	114	4.078276	1.758844	1	1.658	0.204
	221	4.858222	1.478318			
FMT	114	36.0538	2.719245	1	0.978	0.328
	221	36.32322	2.202212			
HR	114	728.4875	98.5392	1	1.642	0.207
	221	778.4627	114.1677			
Mu	114	3.025889	2.982011	1	0.984	0.327
	221	1.75803	3.125248			
PFG	114	29.40923	2.853374	1	2.373	0.13
	221	28.2619	2.580721			
WL	114	6.275726	1.508612	1	0.411	0.525
	221	6.072951	1.486509			
O1_AS_Su	114	29.80231	3.622854	1	1.692	0.2
	221	30.84349	4.902101			
O2_AS_SU	114	29.3758	3.48875	1	3.064	0.087
	221	31.09949	4.781906			
P3_AS_Su	114	30.7602	3.618596	1	1.155	0.288
	221	31.72064	5.41964			
P4_AS_Su	114	30.78243	3.511247	1	1.141	0.291
	221	31.68683	5.111489			

Narrative Version: 114 = Least Just, 221 = Most Just; $\bar{x}$ = Mean; σ = Standard Deviation; df = degrees of freedom; F = Mean Square Factor/Mean Square residual; p = Probability of Significance; $\alpha = 0.05$

Physiological Metrics - Donation*Narrative Version

Physiological Metrics - Interaction (Donated*Narrative Version)			
**Variable**	**df**	**F**	**p**
HRV	1	2.231	0.142
EMGQ	1	5.703	0.021
ENG	1	0.214	0.646
FMT	1	1.265	0.267
HR	1	0	0.985
Mu	1	0.284	0.597
PFG	1	0.663	0.42
WL	1	1.489	0.229
O1_AS_Su	1	0.054	0.817
O2_AS_SU	1	0.168	0.684
P3_AS_Su	1	0.296	0.589
P4_AS_Su	1	0.044	0.835

df = degrees of freedom; F = Mean Square Factor/Mean Square residual; p = Probability of Significance; α = 0.05

Physiological Metrics - Donation*Narrative Version (Post-Hoc)

Physiological Metrics - Interaction Post-Hoc						
**Variable**	**Donated**	**(I) Narrative Version**	**(J) Narrative Version**	**Mean Difference (I-J)**	**Std. Error**	**p**
HRV	N	114	221	0.478	0.543	0.383
	Y	114	221	-0.979	0.81	0.233
EMGQ	N	114	221	0.014	0.056	0.804
	Y	114	221	-.225*	0.083	0.01
ENG	N	114	221	-0.084	0.053	0.123
	Y	114	221	-0.04	0.08	0.622
FMT	N	114	221	0.01	0.08	0.904
	Y	114	221	-0.151	0.119	0.21
HR	N	114	221	-4.13	3.533	0.249
	Y	114	221	-4.009	5.277	0.451
Mu	N	114	221	0.138	0.101	0.178
	Y	114	221	0.042	0.151	0.784
PFG	N	114	221	0.058	0.089	0.517
	Y	114	221	0.188	0.133	0.163
WL	N	114	221	-0.025	0.048	0.605
	Y	114	221	0.081	0.072	0.269
O1_AS_Su	N	114	221	0.138	0.144	0.343
	Y	114	221	0.199	0.216	0.361
O2_AS_SU	N	114	221	0.186	0.155	0.235
	Y	114	221	0.3	0.231	0.2
P3_AS_Su	N	114	221	0.071	0.149	0.635
	Y	114	221	0.217	0.223	0.335
P4_AS_Su	N	114	221	0.114	0.148	0.445
	Y	114	221	0.17	0.221	0.446

Donation: Y = Donated, N = Did not Donate; Narrative Version: 114 = Least Just, 221 = Most Just; p = Probability of Significance; α = 0.05
Note: Mean Difference (J-I) is the inverse value of the Mean Difference (I- J) presented.

ANOVAs - Narrative Segments

Segment 1

Donation and Narrative Version Main Effects on Physiological Metrics

Physiological Metrics - Donation (Segment 1)

IV	DV	Donated	x̄	σ	df	MS	F	p
Donation	EMGQ1	N	0.177	0.165	1	0.028	0.668	0.418
		Y	0.276	0.294				
	ENG1	N	0.415	0.161	1	0.014	0.559	0.459
		Y	0.466	0.148				
	FMT1	N	3.420	0.273	1	0.051	0.726	0.399
		Y	3.385	0.250				
	HR1	N	67.606	9.695	1	42.01	0.424	0.518
		Y	71.101	10.496				
	Mu1	N	0.217	0.303	1	0.046	0.475	0.494
		Y	0.135	0.311				
	PFG1	N	2.668	0.290	1	0.04	0.5	0.483
		Y	2.581	0.258				
	WL2	N	0.569	0.114	1	0.005	0.295	0.59
		Y	0.531	0.142				
	O1_AS1	N	0.477	0.349	1	0.026	0.152	0.698
		Y	0.424	0.498				
	O2_AS1	N	0.582	0.387	1	0.062	0.336	0.565
		Y	0.452	0.504				
	P3_AS1	N	0.341	0.352	1	0.013	0.079	0.78
		Y	0.281	0.475				
	P4_AS1	N	0.392	0.379	1	0.106	0.619	0.435
		Y	0.275	0.456				

Physiological Metrics - Narrative Version (Segment 1)

IV	DV	Version	x̄	σ	df	MS	F	p
Narrative Version	EMGQ1	Least Just	0.197	0.172	1	0.051	1.21	0.277
		Most Just	0.223	0.259				
	ENG1	Least Just	0.393	0.159	1	0.034	1.407	0.242
		Most Just	0.468	0.150				
	FMT1	Least Just	3.393	0.331	1	0.063	0.889	0.351
		Most Just	3.421	0.191				
	HR1	Least Just	66.272	8.825	1	212.2	2.141	0.15
		Most Just	71.071	10.616				
	Mu1	Least Just	0.236	0.338	1	0.043	0.443	0.509
		Most Just	0.146	0.273				
	PFG1	Least Just	2.689	0.304	1	0.066	0.831	0.367
		Most Just	2.592	0.254				
	WL2	Least Just	0.569	0.121	1	0.012	0.787	0.38
		Most Just	0.544	0.128				
	O1_AS1	Least Just	0.501	0.311	1	0.019	0.113	0.738
		Most Just	0.421	0.472				
	O2_AS1	Least Just	0.645	0.380	1	0.337	1.822	0.184
		Most Just	0.441	0.457				
	P3_AS1	Least Just	0.358	0.360	1	0.051	0.309	0.581
		Most Just	0.286	0.428				
	P4_AS1	Least Just	0.402	0.408	1	0.036	0.212	0.647
		Most Just	0.307	0.407				

Donated: Y = Donated, N = Did not Donate; x̄ = Mean; σ = Standard Deviation of Population; df = degrees of freedom; MS = Mean Square; F = Mean Square Factor/Mean Square residual; p = Probability of Significance; α = 0.05

Interaction of Donation and Narrative Version on Physiological Metrics

Physiological Metrics - Interaction (Segment 1)					
IV	DV	df	MS	F	p
Donation*Narrative Version	EMGQ1	1	0.335	7.97	0.007
	ENG1	1	0.004	0.15	0.704
	FMT1	1	0.117	1.65	0.205
	HR1	1	13.18	0.13	0.717
	Mu1	1	0.005	0.05	0.821
	PFG1	1	5E-05	0	0.98
	WL2	1	0.024	1.55	0.22
	O1_AS1	1	0.048	0.28	0.599
	O2_AS1	1	0	0	0.963
	P3_AS1	1	0.008	0.05	0.821
	P4_AS1	1	0.006	0.03	0.855

df = degrees of freedom; MS = Mean Square; F = Mean Square Factor/Mean Square residual; p = Probability of Significance; α = 0.05

Physiological Metrics - Interaction Post-Hoc (Segment 1)						
Variable	Donated	(I) Video Version	(J) Video Version	Mean Difference (I-J)	Std. Error	p
EMGQ1	N	114	221	0.113	0.073	0.129
	Y	114	221	-.258*	0.109	0.023
ENG1	N	114	221	-0.079	0.056	0.166
	Y	114	221	-0.04	0.083	0.631
FMT1	N	114	221	0.029	0.095	0.76
	Y	114	221	-0.19	0.142	0.187
HR1	N	114	221	-3.503	3.547	0.329
	Y	114	221	-5.829	5.299	0.277
Mu1	N	114	221	0.089	0.11	0.427
	Y	114	221	0.044	0.165	0.793
PFG1	N	114	221	0.08	0.101	0.43
	Y	114	221	0.085	0.151	0.576
WL2	N	114	221	-0.014	0.044	0.75
	Y	114	221	0.085	0.066	0.206
O1_AS1	N	114	221	0.114	0.147	0.44
	Y	114	221	-0.025	0.219	0.908
O2_AS1	N	114	221	0.18	0.153	0.248
	Y	114	221	0.192	0.229	0.405
P3_AS1	N	114	221	0.043	0.145	0.769
	Y	114	221	0.102	0.216	0.64
P4_AS1	N	114	221	0.085	0.147	0.565
	Y	114	221	0.037	0.22	0.868

Donation: Y = Donated, N = Did not Donate; Narrative Version: 114 = Least Just, 221 = Most Just; p = Probability of Significance; α = 0.05
Note: Mean Difference (J-I) is the inverse value of the Mean Difference (I- J) presented.

Segment 2

Donation and Narrative Version Main Effects on Physiological Metrics

Physiological Metrics - Donation (Segment 2)

IV	DV	Donated	x̄	σ	df	MS	F	p
Donation	EMGQ2	N	0.100	0.107	1	0.044	1.323	0.256
		Y	0.216	0.296				
	ENG2	N	0.407	0.165	1	0.002	0.086	0.77
		Y	0.438	0.161				
	FMT2	N	3.303	0.263	1	0.025	0.398	0.531
		Y	3.274	0.225				
	HR2	N	67.077	10.614	1	121.1	1.024	0.317
		Y	70.833	11.156				
	Mu2	N	0.223	0.252	1	0.004	0.051	0.822
		Y	0.181	0.358				
	PFG2	N	2.581	0.282	1	0.007	0.089	0.766
		Y	2.518	0.275				
	WL2	N	0.569	0.114	1	0.005	0.295	0.59
		Y	0.531	0.142				
	O1_AS2	N	0.450	0.334	1	0.011	0.058	0.811
		Y	0.400	0.547				
	O2_AS2	N	0.546	0.380	1	0.014	0.07	0.792
		Y	0.448	0.554				
	P3_AS2	N	0.279	0.352	1	0.001	0.006	0.939
		Y	0.244	0.572				
	P4_AS2	N	0.329	0.335	1	0.015	0.087	0.769
		Y	0.259	0.535				

Physiological Metrics - Narrative Version (Segment 2)

IV	DV	Version	x̄	σ	df	MS	F	p
Narrative Version	EMGQ2	Least Just	0.107	0.119	1	0.092	2.776	0.103
		Most Just	0.170	0.248				
	ENG2	Least Just	0.374	0.176	1	0.048	1.854	0.18
		Most Just	0.456	0.142				
	FMT2	Least Just	3.300	0.277	1	0.009	0.142	0.709
		Most Just	3.287	0.227				
	HR2	Least Just	66.387	10.930	1	44.25	0.374	0.544
		Most Just	70.142	10.659				
	Mu2	Least Just	0.266	0.272	1	0.083	0.968	0.33
		Most Just	0.158	0.301				
	PFG2	Least Just	2.621	0.301	1	0.139	1.772	0.19
		Most Just	2.505	0.250				
	WL2	Least Just	0.569	0.121	1	0.012	0.787	0.38
		Most Just	0.544	0.128				
	O1_AS2	Least Just	0.473	0.285	1	0.044	0.244	0.624
		Most Just	0.397	0.507				
	O2_AS2	Least Just	0.615	0.349	1	0.386	1.932	0.171
		Most Just	0.420	0.504				
	P3_AS2	Least Just	0.316	0.333	1	0.148	0.747	0.392
		Most Just	0.224	0.512				
	P4_AS2	Least Just	0.359	0.344	1	0.098	0.553	0.461
		Most Just	0.257	0.465				

Donated: Y = Donated, N = Did not Donate; x̄ = Mean; σ = Standard Deviation of Population; df = degrees of freedom; MS = Mean Square; F = Mean Square Factor/Mean Square residual; p = Probability of Significance; α = 0.05

Interaction of Donation and Narrative Version on Physiological Metrics

Physiological Metrics - Interaction (Segment 2)					
IV	DV	df	MS	F	p
Donation*Narrative Version	EMGQ2	1	0.243	7.33	0.01
	ENG2	1	0.006	0.22	0.642
	FMT2	1	0.09	1.41	0.242
	HR2	1	55.16	0.47	0.498
	Mu2	1	0.01	0.11	0.738
	PFG2	1	0.009	0.12	0.732
	WL2	1	0.024	1.55	0.22
	O1_AS2	1	0	0	0.981
	O2_AS2	1	0.017	0.08	0.773
	P3_AS2	1	0.077	0.39	0.536
	P4_AS2	1	0.005	0.03	0.864

df = egrees of freedom; MS = Mean Square; F = Mean Square Factor/Mean Square residual; p = Probability of Significance; α = 0.05

Physiological Metrics - Interaction Post-Hoc (Segment 2)						
Variable	Donated	(I) Video Version	(J) Video Version	Mean Difference (I-J)	Std. Error	p
EMGQ2	N	114	221	0.061	0.065	0.354
	Y	114	221	-.255*	0.097	0.012
ENG2	N	114	221	-0.095	0.058	0.107
	Y	114	221	-0.046	0.086	0.593
FMT2	N	114	221	0.065	0.09	0.471
	Y	114	221	-0.126	0.134	0.353
HR2	N	114	221	-4.509	3.876	0.251
	Y	114	221	0.248	5.789	0.966
Mu2	N	114	221	0.124	0.105	0.241
	Y	114	221	0.061	0.156	0.699
PFG2	N	114	221	0.089	0.1	0.38
	Y	114	221	0.15	0.149	0.319
WL2	N	114	221	-0.014	0.044	0.75
	Y	114	221	0.085	0.066	0.206
O1_AS2	N	114	221	0.071	0.152	0.643
	Y	114	221	0.064	0.227	0.779
O2_AS2	N	114	221	0.157	0.159	0.328
	Y	114	221	0.24	0.238	0.318
P3_AS2	N	114	221	0.034	0.159	0.83
	Y	114	221	0.212	0.237	0.375
P4_AS2	N	114	221	0.077	0.15	0.61
	Y	114	221	0.123	0.224	0.584

Donation: Y = Donated, N = Did not Donate; Narrative Version: 114 = Least Just, 221 = Most Just; p = Probability of Significance; α = 0.05
Note: Mean Difference (J-I) is the inverse value of the Mean Difference (I- J) presented.

Segment 3

Donation and Narrative Version Main Effects on Physiological Metrics

	Physiological Metrics - Donation (Segment 3)									Physiological Metrics - Narrative Version (Segment 3)							
IV	DV	Donated	x̄	σ	df	MS	F	p	IV	DV	Version	x̄	σ	df	MS	F	p
Donation	EMGQ3	N	0.103	0.106	1	0.024	0.715	0.402	Narrative Version	EMGQ3	Least Just	0.104	0.104	1	0.051	1.484	0.23
		Y	0.186	0.284							Most Just	0.157	0.240				
	ENG3	N	0.398	0.162	1	0.003	0.113	0.739		ENG3	Least Just	0.379	0.163	1	0.021	0.763	0.387
		Y	0.427	0.173							Most Just	0.434	0.165				
	FMT3	N	3.356	0.226	1	0.113	1.84	0.182		FMT3	Least Just	3.332	0.256	1	0.008	0.132	0.718
		Y	3.264	0.274							Most Just	3.318	0.240				
	HR3	N	67.900	10.119	1	15.79	0.157	0.694		HR3	Least Just	66.374	9.124	1	163.4	1.627	0.209
		Y	70.264	9.811							Most Just	70.796	10.404				
	Mu3	N	0.203	0.249	1	0.009	0.102	0.751		Mu3	Least Just	0.236	0.289	1	0.042	0.453	0.505
		Y	0.161	0.383							Most Just	0.147	0.307				
	PFG3	N	2.604	0.266	1	0.008	0.12	0.731		PFG3	Least Just	2.639	0.273	1	0.108	1.582	0.215
		Y	2.544	0.250							Most Just	2.533	0.241				
	WL3	N	0.568	0.133	1	0.003	0.177	0.676		WL3	Least Just	0.569	0.138	1	0.015	0.767	0.386
		Y	0.531	0.154							Most Just	0.543	0.143				
	O1_AS3	N	0.426	0.338	1	0.018	0.088	0.769		O1_AS3	Least Just	0.431	0.312	1	0.005	0.027	0.871
		Y	0.380	0.588							Most Just	0.392	0.527				
	O2_AS3	N	0.528	0.382	1	0.014	0.067	0.797		O2_AS3	Least Just	0.591	0.363	1	0.339	1.587	0.214
		Y	0.433	0.582							Most Just	0.411	0.520				
	P3_AS3	N	0.271	0.342	1	0.008	0.036	0.851		P3_AS3	Least Just	0.289	0.352	1	0.077	0.355	0.554
		Y	0.210	0.622							Most Just	0.215	0.532				
	P4_AS3	N	0.315	0.333	1	0.016	0.077	0.782		P4_AS3	Least Just	0.337	0.359	1	0.091	0.448	0.506
		Y	0.242	0.598							Most Just	0.247	0.502				

Donated: Y = Donated, N = Did not Donate; x̄ = Mean; σ = Standard Deviation of Population; df = degrees of freedom; MS = Mean Square; F = Mean Square Factor/Mean Square residual; p = Probability of Significance; α = 0.05

Interaction of Donation and Narrative Version on Physiological Metrics

Physiological Metrics - Interaction (Segment 3)					
IV	DV	df	MS	F	p
Donation*Narrative Version	EMGQ3	1	0.09	2.65	0.111
	ENG3	1	0.001	0.03	0.87
	FMT3	1	0.027	0.44	0.512
	HR3	1	0.026	0	0.987
	Mu3	1	0.022	0.24	0.628
	PFG3	1	0.004	0.06	0.809
	WL3	1	0.029	1.49	0.229
	O1_AS3	1	0.002	0.01	0.915
	O2_AS3	1	0.023	0.11	0.746
	P3_AS3	1	0.045	0.21	0.651
	P4_AS3	1	0.024	0.12	0.729

Physiological Metrics - Interaction Post-Hoc (Segment 3)						
Variable	Donated	(I) Video Version	(J) Video Version	Mean Difference (I-J)	Std. Error	p
EMGQ3	N	114	221	0.024	0.066	0.714
	Y	114	221	-0.168	0.098	0.094
ENG3	N	114	221	-0.056	0.06	0.356
	Y	114	221	-0.038	0.089	0.672
FMT3	N	114	221	0.023	0.088	0.791
	Y	114	221	-0.081	0.132	0.541
HR3	N	114	221	-4.146	3.572	0.252
	Y	114	221	-4.043	5.335	0.453
Mu3	N	114	221	0.113	0.108	0.302
	Y	114	221	0.018	0.162	0.912
PFG3	N	114	221	0.085	0.093	0.367
	Y	114	221	0.126	0.139	0.371
WL3	N	114	221	-0.016	0.05	0.758
	Y	114	221	0.094	0.075	0.214
O1_AS3	N	114	221	0.039	0.16	0.809
	Y	114	221	0.008	0.239	0.974
O2_AS3	N	114	221	0.138	0.165	0.405
	Y	114	221	0.235	0.246	0.345
P3_AS3	N	114	221	0.021	0.166	0.9
	Y	114	221	0.157	0.248	0.53
P4_AS3	N	114	221	0.046	0.16	0.774
	Y	114	221	0.146	0.239	0.543

df = degrees of freedom; MS = Mean Square; F = Mean Square Factor/Mean Square residual; p = Probability of Significance; α = 0.05

Donation: Y = Donated, N = Did not Donate; Narrative Version: 114 = Least Just, 221 = Most Just; p = Probability of Significance; α = 0.05
Note: Mean Difference (J-I) is the inverse value of the Mean Difference (I- J) presented.

Segment 4

Donation and Narrative Version Main Effects on Physiological Metrics

Physiological Metrics - Donation (Segment 4)

IV	DV	Donated	x̄	σ	df	MS	F	p
Donation	HRV4	N	2.294	1.656	1	10.41	5.204	0.027
		Y	1.375	0.686				
	EMGQ4	N	0.112	0.129	1	0	0.011	0.919
		Y	0.157	0.246				
	ENG4	N	0.390	0.163	1	0.001	0.043	0.836
		Y	0.413	0.159				
	FMT4	N	3.284	0.236	1	0.056	0.943	0.337
		Y	3.244	0.257				
	HR4	N	67.225	10.605	1	87.91	0.794	0.378
		Y	71.257	10.325				
	Mu4	N	0.216	0.248	1	0.002	0.019	0.892
		Y	0.185	0.360				
	PFG4	N	2.627	0.253	1	0	0.007	0.934
		Y	2.581	0.270				
	WL4	N	0.561	0.148	1	0.006	0.26	0.613
		Y	0.520	0.154				
	O1_AS4	N	0.415	0.328	1	0.001	0.008	0.929
		Y	0.402	0.506				
	O2_AS4	N	0.505	0.371	1	0	0.001	0.979
		Y	0.429	0.533				
	P3_AS4	N	0.271	0.334	1	0.007	0.041	0.841
		Y	0.248	0.539				
	P4_AS4	N	0.296	0.334	1	0	0.001	0.977
		Y	0.250	0.531				

Physiological Metrics - Narrative Version (Segment 4)

IV	DV	Version	x̄	σ	df	MS	F	p
Narrative Version	HRV4	Least Just	2.109	1.396	1	0.25	0.125	0.725
		Most Just	1.856	1.527				
	EMGQ4	Least Just	0.096	0.101	1	0.08	2.675	0.109
		Most Just	0.156	0.222				
	ENG4	Least Just	0.371	0.174	1	0.022	0.817	0.371
		Most Just	0.423	0.145				
	FMT4	Least Just	3.257	0.255	1	0.056	0.95	0.335
		Most Just	3.281	0.234				
	HR4	Least Just	65.985	9.840	1	162.9	1.471	0.232
		Most Just	70.957	10.846				
	Mu4	Least Just	0.267	0.276	1	0.091	1.075	0.305
		Most Just	0.151	0.294				
	PFG4	Least Just	2.670	0.281	1	0.146	2.194	0.146
		Most Just	2.559	0.226				
	WL4	Least Just	0.557	0.159	1	0.01	0.423	0.519
		Most Just	0.538	0.144				
	O1_AS4	Least Just	0.462	0.296	1	0.091	0.559	0.459
		Most Just	0.366	0.464				
	O2_AS4	Least Just	0.611	0.360	1	0.699	3.895	0.055
		Most Just	0.362	0.459				
	P3_AS4	Least Just	0.325	0.336	1	0.213	1.21	0.277
		Most Just	0.208	0.468				
	P4_AS4	Least Just	0.362	0.353	1	0.271	1.589	0.214
		Most Just	0.208	0.446				

Donated: Y = Donated, N = Did not Donate; $\bar{x}$ = Mean; σ = Standard Deviation of Population; df = degrees of freedom; MS = Mean Square; F = Mean Square Factor/Mean Square residual; p = Probability of Significance; $\alpha = 0.05$

Interaction of Donation and Narrative Version on Physiological Metrics

Physiological Metrics - Interaction (Segment 4)					
IV	DV	df	MS	F	p
Donation*Narrative Version	HRV4	1	2.46	1.23	0.273
	EMGQ4	1	0.1	3.33	0.075
	ENG4	1	0	0.02	0.901
	FMT4	1	0.108	1.82	0.184
	HR4	1	2.176	0.02	0.889
	Mu4	1	0.026	0.31	0.584
	PFG4	1	0.015	0.23	0.634
	WL4	1	0.03	1.31	0.258
	O1_AS4	1	0	0	0.962
	O2_AS4	1	0.031	0.18	0.678
	P3_AS4	1	0.053	0.3	0.587
	P4_AS4	1	0.012	0.07	0.793

Physiological Metrics - Interaction Post-Hoc (Segment 4)						
Variable	Donated	(I) Video Version	(J) Video Version	Mean Difference (I-J)	Std. Error	p
HRV4	N	114	221	0.342	0.504	0.501
	Y	114	221	-0.663	0.753	0.384
EMGQ4	N	114	221	0.011	0.062	0.865
	Y	114	221	-.192*	0.092	0.043
ENG4	N	114	221	-0.054	0.058	0.36
	Y	114	221	-0.041	0.087	0.641
FMT4	N	114	221	0.029	0.087	0.738
	Y	114	221	-0.181	0.13	0.169
HR4	N	114	221	-4.56	3.75	0.23
	Y	114	221	-3.615	5.601	0.522
Mu4	N	114	221	0.148	0.104	0.16
	Y	114	221	0.045	0.155	0.772
PFG4	N	114	221	0.083	0.092	0.373
	Y	114	221	0.162	0.137	0.244
WL4	N	114	221	-0.024	0.054	0.659
	Y	114	221	0.087	0.08	0.286
O1_AS4	N	114	221	0.103	0.144	0.479
	Y	114	221	0.09	0.215	0.676
O2_AS4	N	114	221	0.211	0.151	0.169
	Y	114	221	0.325	0.226	0.157
P3_AS4	N	114	221	0.074	0.149	0.622
	Y	114	221	0.221	0.223	0.327
P4_AS4	N	114	221	0.132	0.147	0.375
	Y	114	221	0.202	0.22	0.364

df = degrees of freedom; MS = Mean Square; F = Mean Square Factor/Mean Square residual; p = Probability of Significance; α = 0.05

Donation: Y = Donated, N = Did not Donate; Narrative Version: 114 = Least Just, 221 = Most Just; p = Probability of Significance; α = 0.05
Note: Mean Difference (J-I) is the inverse value of the Mean Difference (I- J) presented.

Segment 5

Donation and Narrative Version Main Effects on Physiological Metrics

Physiological Metrics - Donation (Segment 5)

IV	DV	Donated	x̄	σ	df	MS	F	p
Donation	HRV5	N	2.455	1.805	1	9.648	4.08	0.049
		Y	1.626	0.776				
	EMGQ5	N	0.110	0.117	1	0.046	1.084	0.303
		Y	0.244	0.339				
	ENG5	N	0.389	0.179	1	0	0.012	0.913
		Y	0.413	0.158				
	FMT5	N	3.271	0.240	1	0.051	0.888	0.351
		Y	3.223	0.233				
	HR5	N	66.929	10.849	1	68.58	0.618	0.436
		Y	70.218	10.030				
	Mu5	N	0.244	0.258	1	0.004	0.046	0.831
		Y	0.197	0.344				
	PFG5	N	2.637	0.290	1	0.013	0.164	0.687
		Y	2.558	0.261				
	WL5	N	0.576	0.146	1	0.005	0.206	0.652
		Y	0.536	0.154				
	O1_AS5	N	0.485	0.343	1	0.009	0.047	0.828

Physiological Metrics - Narrative Version (Segment 5)

IV	DV	Version	x̄	σ	df	MS	F	p
Narrative Version	HRV5	Least Just	2.267	1.744	1	0.687	0.29	0.593
		Most Just	2.078	1.427				
	EMGQ5	Least Just	0.083	0.109	1	0.273	6.413	0.015
		Most Just	0.222	0.280				
	ENG5	Least Just	0.346	0.180	1	0.071	2.511	0.12
		Most Just	0.443	0.151				
	FMT5	Least Just	3.242	0.275	1	0.035	0.602	0.442
		Most Just	3.266	0.201				
	HR5	Least Just	65.464	10.577	1	123.2	1.111	0.298
		Most Just	70.376	10.242				
	Mu5	Least Just	0.298	0.268	1	0.133	1.597	0.213
		Most Just	0.166	0.295				
	PFG5	Least Just	2.680	0.338	1	0.181	2.324	0.134
		Most Just	2.547	0.203				
	WL5	Least Just	0.574	0.158	1	0.013	0.593	0.445
		Most Just	0.551	0.142				
	O1_AS5	Least Just	0.531	0.278	1	0.117	0.642	0.427

		Y	0.428	0.544							Most Just	0.408	0.511				
	O2_AS5	N	0.582	0.386	1	0.039	0.201	0.656		O2_AS5	Least Just	0.669	0.346	1	0.63	3.242	0.078
		Y	0.443	0.547							Most Just	0.414	0.497				
	P3_AS5	N	0.337	0.328	1	0	0.001	0.971		P3_AS5	Least Just	0.404	0.294	1	0.371	2.008	0.163
		Y	0.277	0.579							Most Just	0.239	0.510				
	P4_AS5	N	0.362	0.325	1	0.016	0.09	0.765		P4_AS5	Least Just	0.411	0.319	1	0.244	1.352	0.251
		Y	0.271	0.565							Most Just	0.259	0.488				

Donated: Y = Donated, N = Did not Donate; $\bar{x}$ = Mean; σ = Standard Deviation of Population; df = degrees of freedom; MS = Mean Square; F = Mean Square Factor/Mean Square residual; p = Probability of Significance; α = 0.05

Interaction of Donation and Narrative Version on Physiological Metrics

Physiological Metrics - Interaction (Segment 5)					
IV	DV	df	MS	F	p
Donation*Narrative Version	HRV5	1	4.234	1.79	0.188
	EMGQ5	1	0.194	4.55	0.038
	ENG5	1	0.01	0.36	0.554
	FMT5	1	0.031	0.54	0.466
	HR5	1	47.67	0.43	0.515
	Mu5	1	0.011	0.13	0.718
	PFG5	1	0.014	0.18	0.677
	WL5	1	0.035	1.57	0.216
	O1_AS5	1	0.003	0.02	0.899
	O2_AS5	1	0.023	0.12	0.733
	P3_AS5	1	0.079	0.43	0.518
	P4_AS5	1	0.025	0.14	0.71

df = degrees of freedom; MS = Mean Square; F = Mean Square Factor/Mean Square residual; p = Probability of Significance; α = 0.05

Donation: Y = Donated, N = Did not Donate; Narrative Version: 114 = Least Just, 221 = Most Just; p = Probability of Significance; α = 0.05

Physiological Metrics - Interaction Post-Hoc (Segment 5)						
Variable	Donated	(I) Video Version	(J) Video Version	Mean Difference (I-J)	Std. Error	p
HRV5	N	114	221	0.394	0.548	0.476
	Y	114	221	-0.924	0.819	0.265
EMGQ5	N	114	221	-0.026	0.074	0.721
	Y	114	221	-.309*	0.11	0.007
ENG5	N	114	221	-0.117	0.06	0.056
	Y	114	221	-0.053	0.089	0.555
FMT5	N	114	221	-0.003	0.086	0.971
	Y	114	221	-0.116	0.128	0.368
HR5	N	114	221	-5.766	3.752	0.131
	Y	114	221	-1.343	5.605	0.812
Mu5	N	114	221	0.15	0.103	0.15
	Y	114	221	0.083	0.154	0.591
PFG5	N	114	221	0.099	0.099	0.326
	Y	114	221	0.174	0.149	0.248
WL5	N	114	221	-0.023	0.053	0.666
	Y	114	221	0.097	0.08	0.23
O1_AS5	N	114	221	0.127	0.152	0.408
	Y	114	221	0.092	0.227	0.687
O2_AS5	N	114	221	0.206	0.157	0.197
	Y	114	221	0.303	0.235	0.204
P3_AS5	N	114	221	0.105	0.153	0.495
	Y	114	221	0.285	0.229	0.22
P4_AS5	N	114	221	0.107	0.151	0.482
	Y	114	221	0.209	0.226	0.36

Note: Mean Difference (J-I) is the inverse value of the Mean Difference (I- J) presented.

Segment 6

Donation and Narrative Version Main Effects on Physiological Metrics

Physiological Metrics - Donation (Segment 6)

IV	DV	Donated	x̄	σ	df	MS	F	p
Donation	HRV6	N	2.807	2.466	1	15.81	3.682	0.061
		Y	1.699	0.884				
	EMGQ6	N	0.119	0.135	1	0.013	0.373	0.545
		Y	0.203	0.276				
	ENG6	N	0.393	0.182	1	0.002	0.066	0.799
		Y	0.414	0.162				
	FMT6	N	3.299	0.246	1	0.073	1.225	0.274
		Y	3.255	0.244				
	HR6	N	67.817	11.255	1	55.71	0.469	0.497
		Y	71.475	10.205				
	Mu6	N	0.239	0.227	1	0.026	0.329	0.569
		Y	0.164	0.364				
	PFG6	N	2.634	0.307	1	0.016	0.163	0.688
		Y	2.624	0.319				
	WL6	N	0.578	0.134	1	0.009	0.438	0.511
		Y	0.531	0.152				
	O1_AS6	N	0.463	0.338	1	0.053	0.279	0.6
		Y	0.346	0.576				
	O2_AS6	N	0.547	0.366	1	0.099	0.505	0.481
		Y	0.350	0.588				
	P3_AS6	N	0.313	0.331	1	0.019	0.091	0.764
		Y	0.209	0.618				
	P4_AS6	N	0.337	0.327	1	0.108	0.581	0.45
		Y	0.172	0.584				

Physiological Metrics - Narrative Version (Segment 6)

IV	DV	Version	x̄	σ	df	MS	F	p
Narrative Version	HRV6	Least Just	2.654	2.523	1	0.228	0.053	0.819
		Most Just	2.218	1.700				
	EMGQ6	Least Just	0.102	0.134	1	0.128	3.58	0.065
		Most Just	0.189	0.235				
	ENG6	Least Just	0.363	0.172	1	0.025	0.825	0.368
		Most Just	0.434	0.172				
	FMT6	Least Just	3.257	0.271	1	0.106	1.789	0.188
		Most Just	3.307	0.219				
	HR6	Least Just	66.316	10.708	1	212.6	1.788	0.188
		Most Just	71.536	10.746				
	Mu6	Least Just	0.286	0.253	1	0.118	1.516	0.225
		Most Just	0.149	0.293				
	PFG6	Least Just	2.694	0.317	1	0.223	2.322	0.135
		Most Just	2.575	0.295				
	WL6	Least Just	0.574	0.146	1	0.011	0.569	0.455
		Most Just	0.552	0.139				
	O1_AS6	Least Just	0.501	0.318	1	0.194	1.013	0.32
		Most Just	0.353	0.509				
	O2_AS6	Least Just	0.637	0.363	1	0.914	4.686	0.036
		Most Just	0.338	0.496				
	P3_AS6	Least Just	0.360	0.298	1	0.302	1.475	0.231
		Most Just	0.204	0.543				
	P4_AS6	Least Just	0.394	0.343	1	0.395	2.123	0.152
		Most Just	0.178	0.487				

Donated: Y = Donated, N = Did not Donate; x̄ = Mean; σ = Standard Deviation of Population; df = degrees of freedom; MS = Mean Square; F = Mean Square Factor/Mean Square residual; p = Probability of Significance; α = 0.05

Interaction of Donation and Narrative Version on Physiological Metrics

Physiological Metrics - Interaction (Segment 6)					
IV	DV	df	MS	F	p
Donation*Narrative Version	HRV6	1	7.35	1.71	0.197
	EMGQ6	1	0.124	3.47	0.069
	ENG6	1	0.024	0.79	0.377
	FMT6	1	0.105	1.76	0.191
	HR6	1	0.108	0	0.976
	Mu6	1	0.018	0.23	0.637
	PFG6	1	0.047	0.49	0.49
	WL6	1	0.035	1.76	0.192
	O1_AS6	1	0.011	0.06	0.811
	O2_AS6	1	0.1	0.51	0.479
	P3_AS6	1	0.088	0.43	0.514
	P4_AS6	1	0.011	0.06	0.808

df = degrees of freedom; MS = Mean Square; F = Mean Square Factor/Mean Square residual; p = Probability of Significance; α = 0.05

Physiological Metrics - Interaction Post-Hoc (Segment 6)						
Variable	Donated	(I) Video Version	(J) Video Version	Mean Difference (I-J)	Std. Error	p
HRV6	N	114	221	0.715	0.738	0.338
	Y	114	221	-1.021	1.103	0.359
EMGQ6	N	114	221	-0.002	0.067	0.98
	Y	114	221	-.227*	0.1	0.029
ENG6	N	114	221	-0.1	0.062	0.113
	Y	114	221	-0.001	0.093	0.992
FMT6	N	114	221	-0.001	0.087	0.992
	Y	114	221	-0.208	0.13	0.116
HR6	N	114	221	-4.564	3.885	0.246
	Y	114	221	-4.775	5.803	0.415
Mu6	N	114	221	0.153	0.1	0.132
	Y	114	221	0.068	0.149	0.652
PFG6	N	114	221	0.082	0.111	0.461
	Y	114	221	0.221	0.165	0.188
WL6	N	114	221	-0.026	0.05	0.61
	Y	114	221	0.094	0.075	0.217
O1_AS6	N	114	221	0.107	0.156	0.495
	Y	114	221	0.175	0.233	0.457
O2_AS6	N	114	221	0.205	0.157	0.199
	Y	114	221	0.407	0.235	0.09
P3_AS6	N	114	221	0.081	0.161	0.619
	Y	114	221	0.271	0.241	0.266
P4_AS6	N	114	221	0.167	0.154	0.282
	Y	114	221	0.235	0.23	0.311

Donation: Y = Donated, N = Did not Donate; Narrative Version: 114 = Least Just, 221 = Most Just; p = Probability of Significance; α = 0.05

Note: Mean Difference (J-I) is the inverse value of the Mean Difference (I- J) presented.

Segment 7

Donation and Narrative Version Main Effects on Physiological Metrics

Physiological Metrics - Donation (Segment 7)

IV	DV	Donated	x̄	σ	df	MS	F	p
Donation	HRV7	N	2.798	2.623	1	13.53	2.695	0.108
		Y	1.801	1.075				
	EMGQ7	N	0.091	0.105	1	0.001	0.053	0.819
		Y	0.132	0.198				
	ENG7	N	0.406	0.167	1	0	0.005	0.944
		Y	0.426	0.149				
	FMT7	N	3.272	0.225	1	0.045	0.88	0.353
		Y	3.228	0.222				
	HR7	N	67.384	10.842	1	0.521	0.004	0.948
		Y	69.247	12.061				
	Mu7	N	0.248	0.287	1	0.048	0.512	0.478
		Y	0.165	0.349				
	PFG7	N	2.652	0.273	1	0.01	0.126	0.724
		Y	2.631	0.302				
	WL7	N	0.573	0.137	1	0.008	0.408	0.526
		Y	0.529	0.147				
	O1_AS7	N	0.467	0.393	1	0.029	0.134	0.716
		Y	0.394	0.556				
	O2_AS7	N	0.548	0.430	1	0.029	0.127	0.723
		Y	0.429	0.559				
	P3_AS7	N	0.332	0.361	1	0.042	0.198	0.659
		Y	0.220	0.597				
	P4_AS7	N	0.360	0.394	1	0.092	0.428	0.516
		Y	0.233	0.561				

Physiological Metrics - Narrative Version (Segment 7)

IV	DV	Version	x̄	σ	df	MS	F	p
Narrative Version	HRV7	Least Just	2.599	2.641	1	0.659	0.131	0.719
		Most Just	2.322	1.881				
	EMGQ7	Least Just	0.076	0.109	1	0.056	2.807	0.101
		Most Just	0.132	0.166				
	ENG7	Least Just	0.381	0.172	1	0.035	1.352	0.251
		Most Just	0.441	0.145				
	FMT7	Least Just	3.250	0.237	1	0.026	0.503	0.482
		Most Just	3.263	0.214				
	HR7	Least Just	64.647	11.280	1	470.4	3.857	0.056
		Most Just	71.023	10.421				
	Mu7	Least Just	0.295	0.309	1	0.089	0.945	0.336
		Most Just	0.152	0.299				
	PFG7	Least Just	2.713	0.305	1	0.249	3.18	0.081
		Most Just	2.584	0.247				
	WL7	Least Just	0.565	0.145	1	0.007	0.336	0.565
		Most Just	0.550	0.139				
	O1_AS7	Least Just	0.504	0.361	1	0.081	0.381	0.54
		Most Just	0.387	0.520				
	O2_AS7	Least Just	0.627	0.410	1	0.492	2.171	0.148
		Most Just	0.400	0.512				
	P3_AS7	Least Just	0.382	0.355	1	0.251	1.191	0.281
		Most Just	0.214	0.521				
	P4_AS7	Least Just	0.394	0.407	1	0.136	0.632	0.431
		Most Just	0.248	0.495				

Donated: Y = Donated, N = Did not Donate; x̄ = Mean; σ = Standard Deviation of Population; df = degrees of freedom; MS = Mean Square; F = Mean Square Factor/Mean Square residual; p = Probability of Significance; α = 0.05

Interaction of Donation and Narrative Version on Physiological Metrics

Physiological Metrics - Interaction (Segment 7)					
IV	DV	df	MS	F	p
Donation*Narrative Version	HRV7	1	5.869	1.17	0.285
	EMGQ7	1	0.05	2.5	0.121
	ENG7	1	0	0.01	0.916
	FMT7	1	0.047	0.91	0.345
	HR7	1	25.02	0.21	0.653
	Mu7	1	0.083	0.88	0.353
	PFG7	1	0.05	0.65	0.426
	WL7	1	0.032	1.6	0.212
	O1_AS7	1	0.014	0.07	0.796
	O2_AS7	1	0.01	0.04	0.834
	P3_AS7	1	0.006	0.03	0.862
	P4_AS7	1	0.001	0.01	0.935

Physiological Metrics - Interaction Post-Hoc (Segment 7)						
Variable	Donated	(I) Video Version	(J) Video Version	Mean Difference (I-J)	Std. Error	p
HRV7	N	114	221	0.516	0.798	0.521
	Y	114	221	-1.036	1.193	0.39
EMGQ7	N	114	221	-0.004	0.05	0.932
	Y	114	221	-0.147	0.075	0.056
ENG7	N	114	221	-0.055	0.058	0.347
	Y	114	221	-0.066	0.086	0.449
FMT7	N	114	221	0.018	0.081	0.826
	Y	114	221	-0.121	0.121	0.322
HR7	N	114	221	-5.345	3.936	0.181
	Y	114	221	-8.549	5.879	0.153
Mu7	N	114	221	0.188	0.109	0.093
	Y	114	221	0.003	0.163	0.984
PFG7	N	114	221	0.088	0.1	0.383
	Y	114	221	0.232	0.149	0.127
WL7	N	114	221	-0.031	0.05	0.541
	Y	114	221	0.083	0.075	0.273
O1_AS7	N	114	221	0.13	0.165	0.435
	Y	114	221	0.053	0.246	0.831
O2_AS7	N	114	221	0.193	0.17	0.262
	Y	114	221	0.257	0.253	0.316
P3_AS7	N	114	221	0.135	0.164	0.414
	Y	114	221	0.186	0.244	0.45
P4_AS7	N	114	221	0.13	0.165	0.435
	Y	114	221	0.106	0.247	0.67

df = degrees of freedom; MS = Mean Square; F = Mean Square Factor/Mean Square residual; p = Probability of Significance; α = 0.05

Donation: Y = Donated, N = Did not Donate; Narrative Version: 114 = Least Just, 221 = Most Just; p = Probability of Significance; α = 0.05
Note: Mean Difference (J-I) is the inverse value of the Mean Difference (I- J) presented.

Segment 8

Donation and Narrative Version Main Effects on Physiological Metrics

Physiological Metrics - Donation (Segment 8)									Physiological Metrics - Narrative Version (Segment 8)								
IV	DV	Donated	x̄	σ	df	MS	F	p	IV	DV	Version	x̄	σ	df	MS	F	p
Donation	HRV8	N	2.785	2.630	1	16.76	3.267	0.077	Narrative Version	HRV8	Least Just	2.419	2.468	1	3.29	0.641	0.427
		Y	1.743	1.206							Most Just	2.428	2.152				
	EMGQ8	N	0.106	0.101	1	0.018	0.507	0.48		EMGQ8	Least Just	0.089	0.089	1	0.148	4.233	0.045
		Y	0.200	0.305							Most Just	0.182	0.255				
	ENG8	N	0.381	0.174	1	0.003	0.126	0.724		ENG8	Least Just	0.350	0.177	1	0.043	1.546	0.22
		Y	0.414	0.153							Most Just	0.430	0.149				
	FMT8	N	3.241	0.205	1	0.036	0.763	0.387		FMT8	Least Just	3.213	0.239	1	0.057	1.215	0.276
		Y	3.213	0.236							Most Just	3.248	0.193				
	HR8	N	66.635	10.784	1	30.94	0.264	0.61		HR8	Least Just	64.876	10.265	1	227.8	1.946	0.17
		Y	69.693	10.995							Most Just	70.190	10.919				
	Mu8	N	0.252	0.237	1	0.024	0.31	0.581		Mu8	Least Just	0.287	0.263	1	0.054	0.695	0.409
		Y	0.191	0.342							Most Just	0.182	0.283				
	PFG8	N	2.630	0.254	1	0	0.003	0.954		PFG8	Least Just	2.671	0.255	1	0.161	2.246	0.141
		Y	2.582	0.294							Most Just	2.562	0.271				
	WL8	N	0.572	0.141	1	0.008	0.408	0.526		WL8	Least Just	0.560	0.148	1	0.003	0.165	0.686
		Y	0.529	0.147							Most Just	0.554	0.142				

O1_AS8	N	0.462	0.348	1	0.005	0.025	0.874
	Y	0.420	0.534				
O2_AS8	N	0.548	0.379	1	0.013	0.066	0.799
	Y	0.440	0.545				
P3_AS8	N	0.334	0.309	1	0.004	0.025	0.876
	Y	0.263	0.566				
P4_AS8	N	0.361	0.321	1	0.023	0.138	0.712
	Y	0.272	0.537				

O1_AS8	Least Just	0.494	0.318	1	0.063	0.346	0.56
	Most Just	0.406	0.491				
O2_AS8	Least Just	0.630	0.366	1	0.546	2.842	0.099
	Most Just	0.405	0.480				
P3_AS8	Least Just	0.378	0.310	1	0.211	1.206	0.278
	Most Just	0.249	0.483				
P4_AS8	Least Just	0.403	0.329	1	0.174	1.027	0.316
	Most Just	0.266	0.459				

Donated: Y = Donated, N = Did not Donate; $\bar{x}$ = Mean; σ = Standard Deviation of Population; df = degrees of freedom; MS = Mean Square; F = Mean Square Factor/Mean Square residual; p = Probability of Significance; $\alpha = 0.05$

Interaction of Donation and Narrative Version on Physiological Metrics

IV	DV	df	MS	F	p
**Physiological Metrics - Interaction (Segment 8)**					
Donation*Narrative Version	HRV8	1	6.003	1.17	0.285
	EMGQ8	1	0.16	4.56	0.038
	ENG8	1	0.007	0.27	0.605
	FMT8	1	0.067	1.44	0.237
	HR8	1	0.193	0	0.968
	Mu8	1	0.033	0.42	0.518
	PFG8	1	0.04	0.57	0.456
	WL8	1	0.036	1.74	0.194
	O1_AS8	1	0.001	0.01	0.947
	O2_AS8	1	0.039	0.21	0.653
	P3_AS8	1	0.052	0.3	0.587
	P4_AS8	1	0.007	0.04	0.841

Physiological Metrics - Interaction Post-Hoc (Segment 8)

Variable	Donated	(I) Video Version	(J) Video Version	Mean Difference (I-J)	Std. Error	p
HRV8	N	114	221	0.204	0.807	0.802
	Y	114	221	-1.366	1.206	0.263
EMGQ8	N	114	221	0.005	0.067	0.944
	Y	114	221	-.251*	0.1	0.015
ENG8	N	114	221	-0.094	0.059	0.12
	Y	114	221	-0.038	0.088	0.666
FMT8	N	114	221	0.007	0.077	0.931
	Y	114	221	-0.159	0.115	0.173
HR8	N	114	221	-4.974	3.855	0.204
	Y	114	221	-4.693	5.758	0.419
Mu8	N	114	221	0.132	0.099	0.189
	Y	114	221	0.016	0.148	0.913
PFG8	N	114	221	0.064	0.095	0.505
	Y	114	221	0.193	0.142	0.183
WL8	N	114	221	-0.042	0.051	0.417
	Y	114	221	0.079	0.076	0.305
O1_AS8	N	114	221	0.09	0.152	0.559
	Y	114	221	0.071	0.228	0.755
O2_AS8	N	114	221	0.173	0.156	0.273
	Y	114	221	0.3	0.233	0.205
P3_AS8	N	114	221	0.074	0.149	0.623
	Y	114	221	0.221	0.223	0.327
P4_AS8	N	114	221	0.107	0.147	0.469
	Y	114	221	0.16	0.219	0.469

df = degrees of freedom; MS = Mean Square; F = Mean Square Factor/Mean Square residual; p = Probability of

Significance; α = 0.05

Donation: Y = Donated, N = Did not Donate; Narrative Version: 114 = Least Just, 221 = Most Just; p = Probability of Significance; α = 0.05
Note: Mean Difference (J-I) is the inverse value of the Mean Difference (I- J) presented.

Segment 9

Donation and Narrative Version Main Effects on Physiological Metrics

Physiological Metrics - Donation (Segment 9)

IV	DV	Donated	x̄	σ	df	MS	F	p
Donation	HRV9	N	2.518	2.265	1	12.39	3.197	0.081
		Y	1.670	1.245				
	EMGQ9	N	0.105	0.098	1	0.038	1.045	0.312
		Y	0.219	0.311				
	ENG9	N	0.402	0.158	1	9E-05	0.004	0.952
		Y	0.410	0.161				
	FMT9	N	3.291	0.224	1	0.071	1.219	0.275
		Y	3.236	0.266				
	HR9	N	68.865	9.577	1	4.37	0.044	0.834
		Y	70.567	10.270				
	Mu9	N	0.232	0.258	1	0.02	0.221	0.64
		Y	0.174	0.368				
	PFG9	N	2.638	0.257	1	0.005	0.064	0.801
		Y	2.571	0.327				
	WL9	N	0.569	0.138	1	0.012	0.576	0.452
		Y	0.519	0.155				

Physiological Metrics - Narrative Version (Segment 9)

IV	DV	Version	x̄	σ	df	MS	F	p
Narrative Version	HRV9	Least Just	2.255	2.305	1	2.698	0.696	0.409
		Most Just	2.196	1.730				
	EMGQ9	Least Just	0.080	0.070	1	0.191	5.283	0.026
		Most Just	0.201	0.261				
	ENG9	Least Just	0.373	0.174	1	0.029	1.121	0.295
		Most Just	0.433	0.139				
	FMT9	Least Just	3.245	0.255	1	0.076	1.312	0.258
		Most Just	3.296	0.224				
	HR9	Least Just	67.817	9.428	1	104.3	1.06	0.309
		Most Just	70.905	9.982				
	Mu9	Least Just	0.268	0.283	1	0.059	0.652	0.424
		Most Just	0.162	0.307				
	PFG9	Least Just	2.651	0.255	1	0.097	1.221	0.275
		Most Just	2.583	0.305				
	WL9	Least Just	0.561	0.147	1	0.006	0.309	0.581
		Most Just	0.543	0.145				

O1_AS9	N	0.467	0.352	1	0.043	0.233	0.632
	Y	0.375	0.535				
O2_AS9	N	0.554	0.387	1	0.047	0.234	0.631
	Y	0.405	0.555				
P3_AS9	N	0.333	0.318	1	0.035	0.191	0.665
	Y	0.223	0.581				
P4_AS9	N	0.356	0.340	1	0.078	0.415	0.523
	Y	0.220	0.567				

O1_AS9	Least Just	0.496	0.307	1	0.093	0.504	0.481
	Most Just	0.381	0.501				
O2_AS9	Least Just	0.634	0.376	1	0.639	3.217	0.08
	Most Just	0.386	0.489				
P3_AS9	Least Just	0.365	0.316	1	0.198	1.067	0.307
	Most Just	0.233	0.500				
P4_AS9	Least Just	0.390	0.355	1	0.209	1.108	0.298
	Most Just	0.237	0.484				

Donated: Y = Donated, N = Did not Donate; $\bar{x}$ = Mean; σ = Standard Deviation of Population; df = degrees of freedom; MS = Mean Square; F = Mean Square Factor/Mean Square residual; p = Probability of Significance; $\alpha = 0.05$

Interaction of Donation and Narrative Version on Physiological Metrics

Physiological Metrics - Interaction (Segment 9)					
IV	DV	df	MS	F	p
Donation*Narrative Version	HRV9	1	9.081	2.34	0.133
	EMGQ9	1	0.106	2.94	0.093
	ENG9	1	0.004	0.15	0.705
	FMT9	1	0.024	0.41	0.526
	HR9	1	11.29	0.12	0.736
	Mu9	1	0.029	0.31	0.578
	PFG9	1	0.133	1.67	0.203
	WL9	1	0.027	1.29	0.263
	O1_AS9	1	1E-05	0	0.994
	O2_AS9	1	0.061	0.31	0.583
	P3_AS9	1	0.059	0.32	0.574
	P4_AS9	1	0.024	0.13	0.724

df = degrees of freedom; MS = Mean Square; F = Mean Square Factor/Mean Square residual; p = Probability of Significance; α = 0.05

Donation: Y = Donated, N = Did not Donate; Narrative Version: 114 = Least Just, 221 = Most Just; p = Probability of Significance; α = 0.05

Physiological Metrics - Interaction Post-Hoc (Segment 9)						
Variable	Donated	(I) Video Version	(J) Video Version	Mean Difference (I-J)	Std. Error	p
HRV9	N	114	221	0.439	0.702	0.535
	Y	114	221	-1.491	1.048	0.162
EMGQ9	N	114	221	-0.036	0.068	0.602
	Y	114	221	-.244*	0.101	0.02
ENG9	N	114	221	-0.074	0.057	0.202
	Y	114	221	-0.035	0.085	0.685
FMT9	N	114	221	-0.039	0.086	0.651
	Y	114	221	-0.138	0.128	0.289
HR9	N	114	221	-2.195	3.534	0.538
	Y	114	221	-4.347	5.279	0.415
Mu9	N	114	221	0.132	0.107	0.225
	Y	114	221	0.024	0.16	0.882
PFG9	N	114	221	-0.017	0.101	0.867
	Y	114	221	0.217	0.15	0.156
WL9	N	114	221	-0.027	0.052	0.606
	Y	114	221	0.078	0.077	0.315
O1_AS9	N	114	221	0.099	0.153	0.522
	Y	114	221	0.097	0.229	0.675
O2_AS9	N	114	221	0.177	0.159	0.271
	Y	114	221	0.335	0.237	0.165
P3_AS9	N	114	221	0.064	0.153	0.677
	Y	114	221	0.22	0.229	0.341
P4_AS9	N	114	221	0.097	0.155	0.534
	Y	114	221	0.196	0.231	0.401

Note: Mean Difference (J-I) is the inverse value of the Mean Difference (I- J) presented.

Segment 10

Donation and Narrative Version Main Effects on Physiological Metrics

Physiological Metrics - Donation (Segment 10)

IV	DV	Donated	x̄	σ	df	MS	F	p
Donation	HRV10	N	2.453	2.054	1	12.41	3.741	0.059
		Y	1.549	1.365				
	EMGQ10	N	0.095	0.095	1	0.025	0.808	0.374
		Y	0.193	0.286				
	ENG10	N	0.422	0.158	1	0.007	0.32	0.574
		Y	0.407	0.147				
	FMT10	N	3.323	0.252	1	0.133	2.009	0.163
		Y	3.239	0.265				
	HR10	N	68.940	11.154	1	10.92	0.084	0.773
		Y	70.716	11.221				
	Mu10	N	0.262	0.267	1	0.012	0.13	0.721
		Y	0.210	0.357				
	PFG10	N	2.658	0.276	1	0.015	0.178	0.675
		Y	2.638	0.326				
	WL10	N	0.581	0.122	1	0.008	0.41	0.525
		Y	0.540	0.162				
	O1_AS10	N	0.503	0.343	1	0.062	0.368	0.547
		Y	0.392	0.507				
	O2_AS10	N	0.572	0.386	1	0.058	0.305	0.584
		Y	0.413	0.539				
	P3_AS10	N	0.369	0.317	1	0.02	0.12	0.73
		Y	0.270	0.540				
	P4_AS10	N	0.373	0.345	1	0.046	0.258	0.614
		Y	0.247	0.543				

Physiological Metrics - Narrative Version (Segment 10)

IV	DV	Version	x̄	σ	df	MS	F	p
Narrative Version	HRV10	Least Just	2.327	1.979	1	0.687	0.207	0.651
		Most Just	1.975	1.811				
	EMGQ10	Least Just	0.079	0.087	1	0.135	4.333	0.043
		Most Just	0.173	0.238				
	ENG10	Least Just	0.377	0.169	1	0.045	1.982	0.166
		Most Just	0.451	0.131				
	FMT10	Least Just	3.282	0.277	1	0.06	0.902	0.347
		Most Just	3.305	0.242				
	HR10	Least Just	68.316	11.268	1	51.67	0.4	0.53
		Most Just	70.653	11.038				
	Mu10	Least Just	0.303	0.283	1	0.076	0.829	0.368
		Most Just	0.192	0.307				
	PFG10	Least Just	2.691	0.274	1	0.176	2.111	0.153
		Most Just	2.616	0.306				
	WL10	Least Just	0.569	0.130	1	0.003	0.156	0.694
		Most Just	0.565	0.145				
	O1_AS10	Least Just	0.533	0.308	1	0.12	0.706	0.405
		Most Just	0.404	0.474				
	O2_AS10	Least Just	0.657	0.375	1	0.699	3.684	0.061
		Most Just	0.393	0.473				
	P3_AS10	Least Just	0.404	0.312	1	0.216	1.285	0.263
		Most Just	0.274	0.471				
	P4_AS10	Least Just	0.434	0.362	1	0.364	2.037	0.16
		Most Just	0.237	0.457				

Donated: Y = Donated, N = Did not Donate; x̄ = Mean; σ = Standard Deviation of Population; df = degrees of freedom; MS = Mean Square; F = Mean Square Factor/Mean Square residual; p = Probability of Significance; α = 0.05

Interaction of Donation and Narrative Version on Physiological Metrics

Physiological Metrics - Interaction (Segment 10)

IV	DV	df	MS	F	p
Donation*Narrative Version	HRV10	1	11.07	3.34	0.074
	EMGQ10	1	0.121	3.88	0.055
	ENG10	1	0.016	0.68	0.414
	FMT10	1	0.067	1.01	0.32
	HR10	1	4.729	0.04	0.849
	Mu10	1	0.019	0.21	0.652
	PFG10	1	0.242	2.9	0.095
	WL10	1	0.037	1.96	0.168
	O1_AS10	1	0	0	0.968
	O2_AS10	1	0.047	0.25	0.623
	P3_AS10	1	0.082	0.49	0.487
	P4_AS10	1	0.016	0.09	0.769

Physiological Metrics - Interaction Post-Hoc (Segment 10)

Variable	Donated	(I) Video Version	(J) Video Version	Mean Difference (I-J)	Std. Error	p
HRV10	N	114	221	0.8	0.649	0.224
	Y	114	221	-1.331	0.969	0.176
EMGQ10	N	114	221	-0.006	0.063	0.92
	Y	114	221	-.229*	0.094	0.019
ENG10	N	114	221	-0.108	0.054	0.051
	Y	114	221	-0.028	0.081	0.728
FMT10	N	114	221	0.005	0.092	0.96
	Y	114	221	-0.161	0.137	0.245
HR10	N	114	221	-1.606	4.052	0.694
	Y	114	221	-2.999	6.053	0.623
Mu10	N	114	221	0.132	0.108	0.226
	Y	114	221	0.044	0.161	0.785
PFG10	N	114	221	-0.023	0.103	0.823
	Y	114	221	0.292	0.154	0.064
WL10	N	114	221	-0.044	0.049	0.372
	Y	114	221	0.079	0.073	0.286
O1_AS10	N	114	221	0.105	0.147	0.476
	Y	114	221	0.116	0.219	0.599
O2_AS10	N	114	221	0.199	0.155	0.207
	Y	114	221	0.337	0.232	0.153
P3_AS10	N	114	221	0.057	0.146	0.699
	Y	114	221	0.241	0.218	0.276
P4_AS10	N	114	221	0.153	0.151	0.315
	Y	114	221	0.233	0.225	0.306

df = degrees of freedom; MS = Mean Square; F = Mean Square Factor/Mean Square residual; p = Probability of Significance; α = 0.05

Donation: Y = Donated, N = Did not Donate; Narrative Version: 114 = Least Just, 221 = Most Just; p = Probability of Significance; α = 0.05
Note: Mean Difference (J-I) is the inverse value of the Mean Difference (I- J) presented.

Segment 11

Donation and Narrative Version Main Effects on Physiological Metrics

Physiological Metrics - Donation (Segment 11)

IV	DV	Donated	x̄	σ	df	MS	F	p
Donation	HRV11	N	2.464	1.882	1	10.23	3.976	0.052
		Y	1.583	0.950				
	EMGQ11	N	0.107	0.099	1	0.006	0.337	0.564
		Y	0.172	0.203				
	ENG11	N	0.406	0.167	1	7E-05	0.003	0.959
		Y	0.419	0.159				
	FMT11	N	3.338	0.251	1	0.146	2.123	0.152
		Y	3.254	0.281				
	HR11	N	67.389	9.820	1	50.7	0.501	0.483
		Y	70.791	10.506				
	Mu11	N	0.236	0.253	1	0.016	0.187	0.667
		Y	0.175	0.359				
	PFG11	N	2.664	0.250	1	0.001	0.009	0.926
		Y	2.610	0.288				
	WL11	N	0.581	0.131	1	0.022	1.147	0.29

Physiological Metrics - Narrative Version (Segment 11)

IV	DV	Version	x̄	σ	df	MS	F	p
Narrative Version	HRV11	Least Just	2.408	1.958	1	0.039	0.015	0.903
		Most Just	1.937	1.353				
	EMGQ11	Least Just	0.095	0.105	1	0.081	4.448	0.041
		Most Just	0.159	0.169				
	ENG11	Least Just	0.370	0.174	1	0.049	1.827	0.183
		Most Just	0.447	0.146				
	FMT11	Least Just	3.283	0.276	1	0.104	1.513	0.225
		Most Just	3.332	0.252				
	HR11	Least Just	66.033	9.039	1	172.5	1.705	0.198
		Most Just	70.814	10.597				
	Mu11	Least Just	0.285	0.285	1	0.108	1.272	0.265
		Most Just	0.153	0.289				
	PFG11	Least Just	2.690	0.257	1	0.131	1.904	0.174
		Most Just	2.606	0.265				
	WL11	Least Just	0.566	0.138	1	0.001	0.069	0.794

	Y	0.523	0.153				
O1_AS11	N	0.454	0.366	1	0.014	0.074	0.786
	Y	0.380	0.535				
O2_AS11	N	0.545	0.405	1	0.04	0.198	0.659
	Y	0.391	0.558				
P3_AS11	N	0.315	0.313	1	0.003	0.019	0.892
	Y	0.234	0.571				
P4_AS11	N	0.340	0.349	1	0.051	0.276	0.602
	Y	0.212	0.555				

	Most Just	0.557	0.144				
O1_AS11	Least Just	0.502	0.332	1	0.169	0.892	0.35
	Most Just	0.363	0.495				
O2_AS11	Least Just	0.640	0.403	1	0.823	4.019	0.051
	Most Just	0.360	0.481				
P3_AS11	Least Just	0.372	0.313	1	0.333	1.892	0.176
	Most Just	0.211	0.484				
P4_AS11	Least Just	0.400	0.382	1	0.351	1.889	0.176
	Most Just	0.203	0.457				

Donated: Y = Donated, N = Did not Donate; $\bar{x}$ = Mean; σ = Standard Deviation of Population; df = degrees of freedom; MS = Mean Square; F = Mean Square Factor/Mean Square residual; p = Probability of Significance; $\alpha = 0.05$

Interaction of Donation and Narrative Version on Physiological Metrics

Physiological Metrics - Interaction (Segment 11)

IV	DV	df	MS	F	p
Donation*Narrative Version	HRV11	1	7.612	2.96	0.092
	EMGQ11	1	0.108	5.88	0.019
	ENG11	1	0.005	0.18	0.675
	FMT11	1	0.057	0.83	0.367
	HR11	1	0.028	0	0.987
	Mu11	1	0.028	0.33	0.571
	PFG11	1	0.109	1.59	0.214
	WL11	1	0.022	1.14	0.29
	O1_AS11	1	0	0	0.968
	O2_AS11	1	0.064	0.31	0.579
	P3_AS11	1	0.077	0.44	0.512
	P4_AS11	1	0.012	0.06	0.801

Physiological Metrics - Interaction Post-Hoc (Segment 11)

Variable	Donated	(I) Video Version	(J) Video Version	Mean Difference (I-J)	Std. Error	p
HRV11	N	114	221	0.821	0.572	0.158
	Y	114	221	-0.947	0.854	0.273
EMGQ11	N	114	221	0.014	0.048	0.778
	Y	114	221	-.196*	0.072	0.009
ENG11	N	114	221	-0.093	0.058	0.118
	Y	114	221	-0.049	0.087	0.579
FMT11	N	114	221	-0.027	0.094	0.776
	Y	114	221	-0.18	0.14	0.204
HR11	N	114	221	-4.26	3.584	0.241
	Y	114	221	-4.152	5.353	0.442
Mu11	N	114	221	0.159	0.104	0.134
	Y	114	221	0.052	0.155	0.739
PFG11	N	114	221	0.01	0.093	0.915
	Y	114	221	0.222	0.139	0.119
WL11	N	114	221	-0.036	0.05	0.472
	Y	114	221	0.06	0.074	0.427
O1_AS11	N	114	221	0.126	0.155	0.421
	Y	114	221	0.137	0.232	0.557
O2_AS11	N	114	221	0.21	0.161	0.2
	Y	114	221	0.372	0.241	0.13
P3_AS11	N	114	221	0.096	0.15	0.524
	Y	114	221	0.274	0.223	0.227
P4_AS11	N	114	221	0.155	0.154	0.319
	Y	114	221	0.225	0.229	0.333

df = degrees of freedom; MS = Mean Square; F = Mean Square Factor/Mean Square residual; p = Probability of Significance; α = 0.05

Donation: Y = Donated, N = Did not Donate; Narrative Version: 114 = Least Just, 221 = Most Just; p = Probability of Significance; $\alpha = 0.05$

Note: Mean Difference (J-I) is the inverse value of the Mean Difference (I- J) presented.

Principal Component Weights

Narrative Reactions

	Narrative Reactions: All Subjects		Narrative Reactions: Donated Only		
**Variable**	**PCA component #1**	**PCA component #2**	**PCA component #1**	**PCA component #2**	**PCA component #3**
Mary_Likeable	-0.290	0.088	0.501	-0.333	0.260
Mary_Sympathetic	-0.346	0.102	-0.464	-0.107	-0.199
Mary_Worthy	-0.300	-0.199	-0.246	-0.492	-0.049
Ramon_Likeable	-0.193	0.068	-0.199	-0.681	-0.104
Ramon_Sympathetic	-0.385	-0.028	-0.088	-0.065	0.181
Ramon_Worthy	-0.502	0.038	-0.138	0.150	0.192
Freight_Likeable	-0.292	0.013	0.075	0.057	0.273
Freight_Sympathetic	0.257	0.697	0.064	0.087	0.411
Freight_Worthy	0.125	-0.628	0.084	0.259	-0.678
Ending_Satisfaction	-0.318	0.232	0.416	-0.116	-0.253
Memory_Percent_Correct	0.026	-0.018	-0.465	0.232	0.209

Personality

	Personality: All Subjects		Personality: Donated Only	
**Variable**	**PCA component #1**	**PCA component #2**	**PCA component #1**	**PCA component #2**
BDI	0.021	-0.016	0.040	0.353
STAI_State	0.064	0.060	0.141	0.225
STAI_Trait	0.012	-0.042	0.131	0.159
IRI	-0.219	0.041	-0.080	-0.010
Extraversion	0.151	-0.495	-0.209	0.212
Friendliness	0.079	0.072	-0.156	0.230
Gregariousness	0.043	0.249	-0.117	0.240
Assertiveness	0.227	0.181	-0.145	0.265
Activity	0.075	0.056	-0.214	-0.003
ExcitementSeeking	0.189	0.022	-0.105	0.157
Cheerfulness	0.046	0.155	-0.165	0.006
Agreeableness	-0.370	-0.064	-0.177	-0.278
Trust	-0.156	-0.010	-0.190	-0.058

Morality	-0.261	-0.022	-0.109	-0.209
Altruism	-0.206	-0.043	-0.172	0.022
Cooperation	-0.286	0.035	-0.165	-0.225
Modesty	-0.281	0.041	0.022	-0.321
Sympathy	-0.331	0.065	-0.146	-0.252
Conscientiousness	-0.066	-0.083	-0.210	0.011
SelfEfficacy	0.138	-0.016	-0.230	0.055
Orderliness	-0.093	0.030	-0.127	0.135
Dutifulness	-0.198	0.039	-0.166	-0.061
AchievementStriving	0.008	0.070	-0.208	0.011
SelfDiscipline	0.059	0.056	-0.160	0.064
Cautiousness	-0.183	0.000	-0.087	-0.282
Neuroticism	-0.126	-0.467	0.205	0.006
Anxiety	-0.152	0.026	0.156	-0.089
Anger	-0.007	0.179	0.196	0.058
Depression	-0.100	0.234	0.201	-0.003
SelfConsciousness	-0.284	0.095	0.180	-0.162
Immoderation	0.030	0.033	0.237	0.092
Vulnerability	-0.115	-0.013	0.225	-0.020
OpenessToExperience	0.012	-0.437	-0.094	-0.042
Imagination	0.003	0.105	0.101	-0.154
Altruistic	-0.128	0.047	-0.141	0.101
Emotionality	-0.025	0.075	0.023	0.038
Adventurousness	0.028	0.156	-0.206	0.055
Intellect	0.037	0.148	-0.161	-0.073
Liberalism	0.092	0.133	0.033	-0.079

Psychophysiology

	Psychohysiology: All Subjects																			Psychophsyiology: Donated Only
**Variable**	**1**	**2**	**3**	**4**	**5**	**6**	**7**	**8**	**9**	**10**	**11**	**12**	**13**	**14**	**15**	**16**	**17**	**18**	**19**	**1**
HRV4	0.013	-0.067	-0.154	-0.030	-0.224	-0.105	-0.016	0.048	0.121	0.060	0.087	0.061	-0.074	-0.018	0.154	-0.001	-0.031	0.032	0.106	0.101
HRV5	0.005	-0.079	-0.174	0.014	-0.094	-0.188	-0.055	0.123	0.064	0.001	0.025	-0.011	0.052	0.126	-0.036	-0.172	0.010	0.106	-0.073	-0.021
HRV6	0.012	-0.077	-0.196	0.029	0.044	-0.048	0.008	-0.031	-0.155	-0.032	-0.048	-0.181	-0.097	-0.033	0.013	-0.083	0.085	-0.027	-0.045	-0.152
HRV7	0.013	-0.065	-0.176	0.010	0.133	0.001	0.018	-0.019	-0.125	-0.086	0.074	-0.080	-0.051	-0.008	0.096	-0.006	0.002	-0.032	-0.037	-0.283

HRV8	0.016	-0.057	-0.189	-0.008	0.109	0.032	0.039	-0.097	-0.069	0.006	-0.024	0.063	0.008	0.006	-0.095	-0.083	-0.003	0.026	-0.037	-0.221
HRV9	0.018	-0.062	-0.135	-0.065	0.053	0.160	0.056	-0.103	-0.028	-0.004	-0.040	0.087	0.155	-0.012	-0.034	0.196	0.010	0.000	0.229	-0.207
HRV10	0.009	-0.068	-0.118	-0.060	-0.086	0.150	-0.025	0.024	0.113	0.116	-0.080	0.076	0.062	0.054	-0.085	-0.016	0.064	-0.056	-0.250	-0.167
HRV11	0.013	-0.024	-0.131	-0.130	-0.045	0.081	-0.012	0.102	-0.007	-0.017	0.113	-0.005	-0.056	-0.038	-0.077	0.067	-0.106	-0.027	0.092	-0.184
EMGQ1	-0.006	-0.051	0.146	0.020	0.033	0.002	0.062	0.076	-0.108	0.187	-0.186	-0.003	0.102	0.050	0.086	0.030	0.143	0.011	0.140	-0.083
EMGQ2	-0.004	-0.125	0.173	-0.064	0.076	-0.061	-0.024	0.081	0.111	0.011	-0.206	-0.046	-0.104	-0.203	-0.133	-0.079	-0.014	-0.025	0.159	-0.113
EMGQ3	0.002	-0.118	0.176	-0.096	-0.011	-0.114	0.026	0.150	0.063	-0.080	0.040	-0.111	0.021	-0.068	0.130	-0.084	0.017	0.162	0.056	-0.125
EMGQ4	-0.001	-0.121	0.077	0.034	0.093	-0.076	0.006	-0.111	0.092	0.167	0.048	-0.097	0.110	-0.037	-0.008	-0.086	-0.199	-0.207	0.071	-0.097
EMGQ5	-0.001	-0.124	0.136	-0.050	-0.080	0.141	0.035	0.100	-0.046	-0.142	0.049	0.176	-0.045	-0.056	-0.114	0.006	-0.044	-0.108	-0.128	-0.010
EMGQ6	0.004	-0.132	0.139	-0.034	-0.108	-0.091	-0.037	-0.032	0.021	0.218	0.064	-0.047	-0.173	-0.022	-0.017	0.227	-0.079	0.088	-0.051	0.027
EMGQ7	-0.004	-0.129	0.091	-0.081	-0.069	0.012	0.064	-0.038	-0.138	-0.124	-0.006	-0.037	0.011	-0.011	0.160	-0.033	0.267	-0.073	0.136	-0.055
EMGQ8	0.005	-0.127	0.135	-0.027	-0.027	-0.057	-0.160	-0.029	-0.107	0.059	0.173	0.024	-0.098	0.145	0.112	0.106	0.026	0.018	-0.089	-0.051
EMGQ9	-0.001	-0.125	0.140	-0.059	0.050	0.025	-0.027	-0.007	0.006	0.053	0.231	0.152	0.077	-0.014	-0.240	-0.229	0.052	0.023	-0.089	-0.097
EMGQ10	-0.008	-0.129	0.067	-0.047	0.008	0.213	-0.107	0.039	0.052	-0.236	-0.003	0.026	0.074	0.016	-0.022	0.107	0.005	0.066	-0.055	-0.034
EMGQ11	-0.014	-0.141	0.088	-0.025	0.039	0.070	0.081	-0.197	0.092	-0.027	-0.312	0.078	0.047	0.181	0.077	-0.052	-0.030	0.054	-0.127	-0.074
ENG1	0.027	-0.121	0.022	-0.157	-0.172	-0.319	-0.244	-0.092	0.163	-0.118	-0.224	-0.097	0.139	-0.036	-0.033	-0.039	-0.030	-0.166	-0.030	0.035
ENG2	0.039	-0.134	0.031	-0.135	-0.076	-0.072	0.132	-0.003	0.037	-0.219	0.059	0.156	-0.124	0.172	-0.071	0.205	-0.087	-0.043	0.182	-0.030
ENG3	0.056	-0.104	-0.005	-0.045	0.112	-0.042	0.231	0.051	0.216	0.034	-0.060	0.131	0.067	-0.018	-0.043	-0.151	0.240	0.147	-0.240	0.008
ENG4	0.042	-0.110	0.045	-0.035	0.017	-0.133	0.286	-0.052	-0.011	-0.188	0.088	0.193	0.068	0.111	0.004	-0.062	-0.157	-0.077	-0.032	-0.038
ENG5	0.033	-0.127	0.041	-0.161	0.016	0.046	0.109	-0.154	0.035	0.175	0.022	-0.196	0.029	0.089	0.327	-0.013	0.007	-0.019	-0.058	-0.068
ENG6	0.049	-0.098	0.035	-0.099	0.069	0.018	0.256	-0.140	0.158	0.108	0.108	0.016	-0.147	-0.187	-0.014	0.035	0.005	-0.033	-0.037	-0.029
ENG7	0.053	-0.115	0.042	-0.141	-0.226	0.123	-0.023	0.199	-0.201	-0.010	-0.126	-0.047	0.216	-0.028	-0.114	-0.052	-0.019	0.042	-0.096	-0.059
ENG8	0.040	-0.101	0.032	-0.078	0.031	0.189	-0.265	-0.082	-0.126	-0.066	0.004	0.029	-0.037	-0.066	0.031	-0.032	-0.005	0.018	-0.011	-0.030
ENG9	0.048	-0.107	0.032	-0.057	0.076	0.065	-0.190	0.019	-0.065	0.124	-0.028	-0.028	-0.082	0.000	0.041	0.015	0.028	-0.014	0.106	-0.062
ENG10	0.035	-0.090	0.002	-0.045	-0.028	0.136	-0.211	0.018	-0.060	0.187	0.118	-0.022	-0.077	0.121	-0.223	0.073	0.013	-0.098	0.079	0.000
ENG11	0.041	-0.116	-0.005	-0.018	0.061	0.035	-0.097	0.146	0.030	0.064	-0.004	-0.031	0.071	-0.073	0.046	0.036	-0.011	0.184	0.092	0.001
MT1	-0.014	0.080	-0.115	0.072	-0.227	0.209	-0.047	-0.007	0.395	-0.139	-0.176	-0.137	-0.137	-0.075	0.059	0.050	-0.094	-0.086	0.059	0.112
MT2	-0.018	0.069	-0.125	0.082	-0.092	0.128	-0.212	-0.061	-0.045	-0.252	0.005	0.061	-0.258	0.036	-0.065	-0.099	-0.105	0.218	-0.102	0.128
MT3	-0.023	0.063	-0.101	0.070	-0.268	-0.018	0.149	-0.211	-0.059	0.035	-0.022	-0.109	-0.038	0.136	-0.242	0.096	0.322	-0.020	0.077	0.198
MT4	-0.033	0.066	-0.125	0.070	0.024	-0.040	-0.066	-0.046	0.082	0.120	0.076	0.041	0.086	0.129	0.027	-0.144	-0.047	0.015	0.016	0.136
MT5	-0.022	0.074	-0.164	0.069	0.027	-0.028	-0.139	0.088	0.100	0.090	-0.137	0.190	0.116	-0.242	0.072	-0.093	-0.009	-0.088	-0.018	0.163
MT6	-0.012	0.052	-0.044	0.054	-0.053	0.132	-0.072	-0.049	-0.145	0.140	0.197	0.006	0.055	0.023	0.104	-0.038	-0.111	-0.117	0.095	0.170
MT7	-0.042	0.064	-0.129	0.055	0.091	0.091	0.109	0.074	-0.291	-0.034	-0.134	0.166	0.024	-0.025	0.018	0.012	-0.015	0.000	-0.075	0.121
MT8	-0.016	0.040	-0.052	0.016	0.035	0.002	0.176	-0.019	-0.070	-0.024	0.063	-0.254	0.157	-0.007	-0.133	0.124	-0.175	0.026	-0.093	0.091
MT9	-0.031	0.029	-0.132	0.012	0.040	-0.125	0.089	0.136	-0.010	0.001	0.006	-0.033	-0.012	-0.039	0.078	0.100	0.214	0.016	-0.069	0.142
MT10	-0.027	0.018	-0.143	0.073	0.190	-0.127	-0.026	0.222	-0.028	-0.085	0.113	-0.195	0.075	0.044	0.052	-0.030	-0.089	-0.116	-0.051	0.078
MT11	-0.024	0.044	-0.138	0.001	0.100	-0.088	0.005	-0.163	-0.015	0.069	-0.018	0.151	-0.090	-0.043	0.019	-0.020	0.141	0.135	0.165	0.101

HR1	-0.001	-0.084	0.062	0.004	-0.139	-0.012	-0.046	0.012	0.009	-0.009	0.042	-0.100	0.303	-0.051	-0.204	-0.015	-0.049	0.058	0.022	0.026
HR2	0.001	-0.104	0.091	0.030	0.026	-0.092	-0.129	-0.224	-0.040	-0.053	-0.013	0.096	0.003	-0.042	0.063	0.042	0.026	-0.045	-0.148	-0.056
HR3	0.001	-0.096	0.066	0.029	-0.049	0.023	-0.065	-0.113	-0.025	-0.035	0.026	0.039	0.029	-0.040	-0.002	-0.051	-0.045	-0.082	0.084	-0.006
HR4	-0.002	-0.094	0.083	0.040	-0.004	0.053	-0.007	-0.053	0.029	-0.031	-0.014	0.108	-0.065	-0.045	0.101	-0.064	0.021	0.019	-0.010	0.010
HR5	0.008	-0.106	0.086	0.047	0.009	0.078	0.031	0.021	-0.045	-0.021	0.006	0.117	-0.077	-0.010	0.056	0.011	0.086	0.056	0.022	-0.027
HR6	0.009	-0.091	0.101	0.071	0.016	0.119	0.075	0.000	0.056	0.070	0.150	-0.103	-0.036	0.033	-0.049	-0.058	0.023	0.016	-0.020	-0.033
HR7	0.005	-0.085	0.040	0.064	0.004	0.154	0.192	0.156	-0.007	0.121	-0.022	-0.079	-0.013	0.013	-0.038	-0.045	-0.184	0.089	0.062	-0.003
HR8	0.014	-0.104	0.086	0.068	0.045	0.074	0.083	0.083	0.066	-0.024	0.038	-0.152	0.001	0.022	0.030	0.067	0.053	-0.043	0.060	-0.039
HR9	0.019	-0.093	0.036	-0.063	0.028	-0.142	-0.090	0.094	-0.015	-0.022	-0.057	0.003	-0.088	0.097	-0.089	0.082	-0.048	-0.047	0.050	-0.068
HR10	0.015	-0.072	0.028	-0.063	0.058	-0.208	-0.123	0.036	-0.069	-0.009	-0.065	-0.028	-0.030	0.014	-0.005	0.036	0.022	-0.034	-0.016	-0.024
HR11	0.004	-0.112	0.081	0.032	0.087	0.027	0.035	-0.030	0.049	-0.052	-0.058	0.111	-0.083	0.036	0.087	0.093	0.038	-0.053	-0.041	-0.062
Mu1	0.111	-0.033	0.045	0.102	-0.063	0.076	0.093	-0.093	-0.149	0.099	-0.025	0.006	-0.016	-0.109	-0.058	0.033	0.071	-0.078	0.031	0.054
Mu2	0.128	-0.017	0.047	0.121	0.097	-0.040	0.095	-0.015	-0.015	0.076	0.011	0.005	0.065	-0.031	-0.033	0.201	-0.051	-0.029	-0.180	0.031
Mu3	0.130	-0.012	0.041	0.133	0.149	0.034	-0.128	-0.011	-0.002	0.011	0.099	0.069	0.000	-0.032	0.029	-0.041	-0.076	-0.068	-0.040	0.002
Mu4	0.130	-0.009	0.063	0.085	0.075	0.060	0.017	-0.017	-0.118	-0.128	-0.040	0.037	0.022	-0.041	-0.069	0.061	-0.004	-0.029	0.044	0.011
Mu5	0.130	-0.009	0.068	0.090	0.040	0.007	0.032	-0.226	-0.060	-0.035	0.035	-0.150	-0.034	0.072	-0.074	-0.048	-0.145	0.004	0.069	0.000
Mu6	0.131	-0.013	0.012	0.091	0.029	-0.127	0.099	0.012	-0.010	-0.146	0.095	0.037	0.129	-0.074	-0.030	0.094	-0.035	0.096	0.039	-0.021
Mu7	0.129	-0.007	0.032	0.183	-0.057	-0.048	-0.058	-0.008	0.167	0.043	0.065	0.004	0.043	0.067	0.073	-0.009	0.057	-0.058	-0.156	0.052
Mu8	0.132	0.000	0.035	0.149	-0.010	0.046	-0.043	-0.063	-0.034	0.041	0.030	0.122	-0.009	-0.042	0.027	0.019	-0.018	-0.013	0.010	0.045
Mu9	0.132	0.001	0.078	0.151	-0.054	0.027	-0.043	-0.020	-0.044	-0.017	0.019	-0.019	0.011	-0.020	0.115	0.045	0.146	0.038	-0.085	0.005
Mu10	0.129	0.008	0.082	0.156	-0.123	0.027	0.079	-0.050	-0.029	0.028	0.042	0.019	-0.060	-0.023	-0.012	-0.176	0.045	0.060	-0.017	0.054
Mu11	0.130	0.002	0.074	0.151	-0.055	0.059	0.007	0.128	-0.056	-0.049	0.042	0.017	0.079	0.069	0.085	-0.039	-0.047	-0.012	0.108	0.024
PFG1	0.017	0.163	0.147	-0.013	-0.267	-0.178	-0.001	0.078	0.047	0.042	0.175	0.227	0.026	-0.073	-0.040	0.149	-0.059	0.086	-0.060	-0.169
PFG2	0.015	0.158	0.181	-0.055	-0.046	-0.140	-0.084	0.017	-0.222	-0.058	-0.074	0.054	0.021	-0.177	0.021	-0.047	-0.007	-0.088	0.002	-0.228
PFG3	0.024	0.167	0.194	-0.049	-0.049	-0.041	0.006	-0.106	-0.051	0.056	-0.089	-0.119	0.001	-0.085	-0.081	-0.051	0.008	-0.176	-0.064	-0.224
PFG4	0.003	0.183	0.125	0.006	0.185	-0.031	-0.065	-0.156	0.099	-0.035	0.189	-0.133	0.093	0.042	0.039	0.000	-0.048	0.189	-0.012	-0.208
PFG5	0.013	0.191	0.124	-0.017	0.142	-0.055	-0.138	0.110	0.000	0.010	-0.026	0.131	0.113	0.251	0.102	0.183	0.032	-0.076	-0.023	-0.189
PFG6	0.011	0.174	0.196	-0.042	0.155	-0.107	-0.041	0.012	0.027	0.090	-0.085	0.063	-0.192	0.059	-0.030	-0.046	0.004	0.104	-0.042	-0.173
PFG7	-0.004	0.189	0.140	-0.123	0.135	0.070	-0.016	-0.051	-0.004	-0.122	-0.140	-0.068	-0.018	-0.011	-0.119	0.039	0.038	-0.086	0.044	-0.213
PFG8	0.005	0.176	0.147	-0.138	0.000	0.072	0.090	0.031	0.161	-0.133	-0.068	-0.085	-0.068	0.255	-0.121	0.003	0.040	-0.136	0.059	-0.165
PFG9	0.004	0.170	0.105	-0.172	-0.023	0.065	0.137	0.060	0.035	0.185	-0.078	0.119	0.095	0.070	0.093	-0.174	-0.027	0.026	0.245	-0.063
PFG10	0.006	0.156	0.156	-0.155	-0.012	0.178	0.002	0.036	0.037	-0.002	0.117	-0.052	0.078	0.024	0.183	-0.034	-0.075	0.165	-0.154	-0.135
PFG11	-0.006	0.178	0.135	-0.123	-0.036	0.095	0.028	0.092	0.001	-0.072	0.165	-0.016	-0.179	-0.305	-0.029	-0.029	0.104	-0.046	-0.076	-0.098
WL1	0.039	0.137	-0.054	0.089	-0.097	0.037	0.048	0.057	0.136	0.012	0.032	0.086	-0.042	0.053	0.038	0.161	0.025	-0.255	-0.148	0.050
WL2	0.041	0.119	-0.017	0.014	-0.012	0.035	0.074	0.035	-0.055	-0.096	0.044	0.022	0.085	0.043	0.117	-0.027	0.211	-0.068	0.112	0.062
WL3	0.035	0.126	0.003	0.000	0.038	0.025	-0.032	0.016	0.050	-0.143	0.145	-0.091	-0.102	-0.016	0.098	0.014	0.110	-0.007	0.047	0.069
WL4	0.039	0.138	0.013	-0.013	-0.036	-0.012	-0.028	-0.154	0.065	-0.163	0.187	-0.028	0.096	-0.041	-0.002	-0.356	0.139	-0.112	0.051	0.016

WL5	0.037	0.127	0.005	-0.021	-0.024	-0.084	-0.047	-0.039	-0.054	0.003	-0.092	-0.086	-0.104	0.061	0.063	0.127	-0.058	0.160	-0.102	0.059
WL6	0.031	0.138	0.012	-0.021	-0.008	-0.130	-0.027	-0.091	-0.019	0.087	-0.010	-0.001	0.045	0.045	-0.145	0.000	0.084	0.159	0.143	0.014
WL7	0.033	0.138	-0.036	0.011	-0.060	0.017	-0.044	-0.098	0.031	-0.019	0.002	-0.051	0.154	-0.048	-0.049	0.124	0.007	0.116	0.067	0.079
WL8	0.032	0.144	-0.016	-0.047	0.046	0.052	0.042	-0.025	0.003	0.046	-0.057	-0.025	0.032	-0.111	-0.142	-0.007	-0.186	0.130	-0.009	0.038
WL9	0.034	0.128	-0.025	-0.044	-0.002	0.046	-0.015	0.004	-0.026	0.120	-0.157	0.017	-0.031	-0.043	0.073	0.040	-0.064	0.213	0.022	0.047
WL10	0.033	0.128	0.002	-0.015	-0.038	0.075	0.068	0.082	-0.079	0.089	-0.073	0.038	-0.053	0.170	-0.142	-0.118	-0.029	-0.081	-0.180	0.048
WL11	0.040	0.124	-0.029	-0.009	0.030	0.061	0.074	0.079	0.010	0.109	-0.067	0.180	0.060	-0.097	0.116	0.094	-0.156	-0.282	-0.014	0.046
O1_AS1	0.116	-0.010	-0.048	-0.101	-0.077	0.074	0.007	-0.004	0.029	-0.082	-0.014	0.006	-0.045	0.028	0.122	-0.035	-0.062	0.034	0.079	0.052
O1_AS2	0.132	-0.004	-0.061	-0.104	0.052	0.047	0.057	0.074	0.024	-0.019	0.033	-0.052	-0.134	0.069	-0.041	-0.070	-0.055	-0.031	0.005	0.018
O1_AS3	0.133	-0.004	-0.068	-0.075	0.208	0.038	-0.075	0.039	0.122	0.041	0.026	0.081	-0.010	0.089	-0.141	-0.023	0.035	-0.022	0.115	-0.022
O1_AS4	0.133	0.000	-0.046	-0.105	0.065	0.004	0.026	0.007	-0.023	-0.153	-0.029	0.064	0.093	-0.026	0.050	-0.010	0.028	0.028	-0.030	0.015
O1_AS5	0.131	0.002	-0.064	-0.079	0.092	-0.004	0.036	-0.048	0.023	-0.039	-0.014	-0.049	0.022	-0.105	-0.030	0.090	0.006	-0.083	-0.029	0.019
O1_AS6	0.131	0.002	-0.103	-0.124	0.045	-0.093	0.065	0.026	-0.029	-0.033	-0.025	0.043	-0.180	-0.100	0.032	-0.020	-0.038	-0.009	-0.038	-0.019
O1_AS7	0.134	-0.003	-0.049	-0.065	0.014	0.002	-0.028	0.050	0.112	0.003	0.107	-0.011	0.076	-0.049	0.000	0.097	0.123	0.054	-0.004	0.011
O1_AS8	0.128	-0.003	-0.069	-0.111	0.054	-0.019	-0.009	0.074	-0.010	0.019	-0.006	-0.048	0.059	-0.115	-0.044	-0.030	0.059	0.000	-0.062	0.025
O1_AS9	0.134	0.005	-0.041	-0.070	0.023	-0.009	0.008	0.056	-0.032	-0.001	-0.009	-0.122	-0.021	0.024	0.080	0.006	0.035	-0.095	-0.028	-0.010
O1_AS10	0.133	0.013	-0.050	-0.067	-0.051	-0.054	0.014	0.064	-0.061	0.042	-0.037	-0.011	-0.119	0.158	0.052	-0.132	-0.097	-0.037	0.022	0.018
O1_AS11	0.133	0.005	-0.047	-0.075	-0.016	-0.046	0.026	0.129	-0.002	-0.039	0.036	-0.044	0.048	0.038	-0.003	0.001	0.000	0.050	-0.003	0.022
O2_AS1	0.125	0.003	-0.017	-0.022	-0.100	0.072	-0.027	-0.109	0.014	-0.013	0.032	0.007	-0.015	0.018	0.140	-0.092	-0.061	-0.089	0.069	0.017
O2_AS2	0.136	0.005	-0.023	-0.018	-0.025	0.039	0.020	0.003	0.020	-0.015	0.039	0.045	-0.045	0.107	-0.074	-0.084	0.033	0.018	0.067	0.014
O2_AS3	0.137	0.007	-0.029	0.010	0.090	0.026	-0.071	-0.055	0.123	0.043	0.001	0.110	0.045	0.112	-0.089	0.042	0.011	0.007	0.030	-0.014
O2_AS4	0.137	0.013	-0.010	-0.024	-0.045	-0.001	-0.011	-0.118	0.000	-0.120	-0.082	0.063	0.137	0.025	0.046	0.028	-0.062	0.096	0.009	0.007
O2_AS5	0.134	0.019	-0.035	0.004	-0.014	0.017	0.031	-0.181	0.007	-0.029	-0.046	-0.002	0.052	-0.064	0.025	0.099	-0.099	-0.039	-0.031	0.009
O2_AS6	0.134	0.020	-0.058	-0.017	-0.024	-0.129	0.068	-0.081	-0.061	0.011	-0.010	0.097	-0.080	-0.063	-0.031	0.061	-0.019	0.044	0.132	-0.010
O2_AS7	0.134	0.000	-0.016	0.017	-0.049	0.006	-0.039	-0.056	0.085	0.039	0.062	0.065	0.061	-0.001	-0.037	0.111	0.068	0.106	-0.017	0.016
O2_AS8	0.132	0.014	-0.031	-0.016	-0.039	0.033	-0.060	-0.055	-0.022	0.079	-0.046	-0.040	0.053	-0.083	-0.073	0.043	0.103	-0.032	-0.100	0.021
O2_AS9	0.137	0.019	-0.017	0.019	-0.053	0.009	-0.011	-0.058	-0.063	0.048	-0.046	-0.032	-0.020	0.085	0.015	0.008	0.028	0.028	-0.050	-0.003
O2_AS10	0.136	0.016	-0.018	0.024	-0.133	-0.082	0.007	-0.092	-0.088	0.085	-0.062	0.070	-0.030	0.073	0.010	-0.174	-0.097	-0.024	-0.024	0.015
O2_AS11	0.134	0.016	-0.017	0.010	-0.098	-0.044	-0.026	0.021	-0.073	-0.004	0.030	0.019	0.048	0.089	-0.001	0.065	-0.016	-0.004	-0.002	0.017
P3_AS1	0.119	-0.010	-0.034	-0.133	-0.126	0.081	0.000	-0.031	0.030	0.063	-0.001	0.035	0.044	-0.106	0.088	0.014	-0.020	0.136	-0.037	0.015
P3_AS2	0.125	-0.003	-0.036	-0.137	0.026	0.009	-0.006	0.027	0.016	0.058	-0.025	-0.026	-0.076	0.064	-0.077	0.044	0.023	0.118	-0.100	-0.010
P3_AS3	0.129	-0.002	-0.049	-0.105	0.153	0.057	-0.156	-0.010	0.036	0.075	0.028	0.040	-0.007	0.022	-0.057	-0.056	0.046	-0.081	0.062	-0.049
P3_AS4	0.129	0.007	-0.015	-0.138	0.043	0.025	-0.030	-0.026	-0.077	-0.073	0.009	0.080	0.102	0.000	0.001	-0.016	0.063	0.037	-0.012	-0.033
P3_AS5	0.132	0.008	-0.036	-0.135	0.069	0.018	0.027	-0.143	-0.024	-0.001	0.023	-0.080	0.060	0.025	-0.013	0.037	-0.016	-0.019	-0.098	-0.046
P3_AS6	0.126	0.007	-0.064	-0.165	0.049	-0.076	0.038	-0.006	-0.048	0.034	0.080	0.001	-0.143	-0.121	-0.039	0.008	-0.061	-0.049	-0.080	-0.069
P3_AS7	0.137	0.006	-0.030	-0.085	-0.020	0.012	-0.024	0.001	0.075	0.041	0.110	0.070	0.071	-0.015	0.036	0.030	0.037	-0.068	0.047	-0.028
P3_AS8	0.133	0.007	-0.051	-0.148	0.006	0.029	-0.052	0.001	-0.012	0.023	0.018	-0.075	-0.004	-0.082	0.020	0.015	0.027	-0.017	0.016	-0.016

P3_AS9	0.136	0.013	-0.011	-0.102	-0.040	-0.007	-0.009	0.030	-0.043	0.030	-0.011	-0.083	0.012	0.019	0.127	0.043	0.094	-0.110	-0.047	-0.050
P3_AS10	0.133	0.023	-0.015	-0.109	-0.121	-0.004	0.019	0.046	-0.058	0.078	-0.027	-0.012	-0.133	0.088	0.027	-0.112	-0.079	0.004	0.006	-0.010
P3_AS11	0.135	0.016	-0.010	-0.113	-0.065	-0.031	0.013	0.087	-0.028	-0.001	0.028	-0.053	0.100	0.052	0.033	0.018	-0.020	0.033	0.005	-0.031
P4_AS1	0.123	-0.020	0.028	0.140	-0.054	0.068	0.008	0.070	0.091	0.040	-0.055	0.002	0.006	-0.122	0.035	0.021	0.069	0.074	0.185	0.091
P4_AS2	0.136	-0.015	0.032	0.103	0.042	0.008	0.002	0.123	0.070	-0.022	-0.024	-0.001	-0.069	0.012	-0.084	-0.048	0.083	-0.012	0.047	0.046
P4_AS3	0.136	-0.013	0.030	0.133	0.131	0.014	-0.098	0.064	0.125	0.010	-0.057	0.032	-0.021	0.058	-0.016	0.002	0.025	0.021	0.025	0.015
P4_AS4	0.136	-0.006	0.054	0.101	0.049	0.005	-0.002	0.008	-0.002	-0.142	-0.155	-0.025	0.057	0.030	0.046	-0.086	-0.091	0.039	-0.009	0.033
P4_AS5	0.135	-0.002	0.031	0.118	0.079	0.045	0.038	-0.046	0.007	-0.058	-0.125	-0.085	-0.056	-0.053	0.041	0.002	-0.102	0.140	-0.005	0.038
P4_AS6	0.134	-0.001	0.008	0.104	0.055	-0.190	0.079	0.040	0.013	-0.043	0.023	0.009	-0.071	-0.097	-0.075	-0.010	-0.079	-0.020	0.131	0.000
P4_AS7	0.134	-0.018	0.019	0.154	-0.012	0.003	-0.032	0.051	0.127	0.010	-0.012	-0.010	0.024	-0.036	-0.014	-0.003	-0.049	-0.039	0.016	0.051
P4_AS8	0.137	-0.003	0.034	0.118	0.017	0.068	-0.065	0.082	0.010	0.049	-0.062	-0.130	-0.014	-0.076	-0.110	0.020	0.154	-0.037	-0.029	0.043
P4_AS9	0.137	0.006	0.051	0.131	-0.002	-0.003	0.007	0.077	-0.035	0.009	-0.070	-0.081	-0.102	0.097	0.010	-0.001	0.131	-0.025	-0.060	0.003
P4_AS10	0.135	0.003	0.056	0.144	-0.106	-0.082	0.057	0.030	-0.054	0.042	-0.050	-0.054	-0.072	-0.083	0.026	-0.089	-0.075	-0.003	-0.056	0.048
P4_AS11	0.135	0.003	0.048	0.132	-0.048	-0.026	0.004	0.180	-0.081	-0.072	0.002	-0.022	0.016	0.095	-0.022	-0.010	-0.025	0.022	0.092	0.034

All Metrics Combined

	All Variables: All Subjects						All Variables: Donated Only
**Variable**	**PCA component #1**	**PCA component #2**	**PCA component #3**	**PCA component #4**	**PCA component #5**	**PCA component #6**	**PCA component #1**
Mary_Likeable	-0.027	0.116	0.082	0.173	-0.031	-0.074	-0.016
Mary_Sympathetic	-0.019	0.048	-0.049	0.030	0.035	-0.021	-0.011
Mary_Worthy	-0.026	0.040	-0.155	-0.038	0.087	0.174	0.128
Ramon_Likeable	-0.018	0.075	-0.205	-0.016	0.117	-0.029	0.095
Ramon_Sympathetic	-0.061	0.001	-0.034	0.031	-0.039	-0.001	-0.026
Ramon_Worthy	-0.086	0.013	-0.130	-0.168	-0.196	0.030	0.059
Freight_Likeable	-0.001	0.085	0.102	0.097	0.130	-0.065	0.082
Freight_Sympathetic	0.044	-0.212	-0.113	0.099	0.222	-0.104	0.092
Freight_Worthy	0.044	-0.107	-0.126	-0.020	0.017	0.101	0.066
Ending_Satisfaction	-0.067	-0.067	-0.038	-0.185	0.190	-0.034	0.104
Memory_Percent_Correct	-0.035	-0.103	-0.164	0.057	-0.334	0.120	0.146
BDI	0.041	0.039	0.031	-0.128	0.038	0.039	-0.092
STAI_State	0.059	0.001	-0.162	0.115	-0.013	-0.103	0.005
STAI_Trait	0.032	-0.041	-0.144	0.149	0.078	0.034	0.052
IRI	0.013	-0.187	0.054	0.046	0.000	-0.050	-0.110
Extraversion	0.109	0.095	0.104	0.056	0.037	0.030	-0.123

Friendliness	0.109	0.104	0.118	-0.154	-0.006	-0.218	-0.100
Gregariousness	0.094	0.004	0.175	-0.017	0.044	0.103	-0.089
Assertiveness	0.106	0.151	-0.165	0.148	0.043	-0.022	-0.053
Activity	0.058	0.179	-0.027	-0.015	0.032	0.037	-0.019
ExcitementSeeking	0.035	0.037	0.136	0.039	-0.050	0.042	-0.129
Cheerfulness	0.067	0.012	0.123	0.265	-0.062	0.160	-0.130
Agreeableness	0.012	-0.155	0.005	0.001	-0.050	0.019	0.038
Trust	0.039	-0.059	0.069	-0.078	-0.116	-0.168	0.005
Morality	0.022	-0.095	-0.001	0.203	-0.062	-0.080	0.022
Altruism	0.090	-0.126	0.019	-0.066	-0.029	0.028	-0.070
Cooperation	-0.020	-0.052	0.085	-0.213	-0.077	0.011	-0.039
Modesty	-0.051	-0.123	0.139	0.019	-0.064	0.076	0.091
Sympathy	-0.025	-0.171	-0.135	0.148	0.059	0.138	0.060
Conscientiousness	0.086	0.095	-0.045	0.009	0.053	0.008	-0.037
SelfEfficacy	0.088	0.163	-0.067	-0.002	0.056	0.073	-0.067
Orderliness	0.080	0.031	-0.024	0.120	0.005	0.062	-0.019
Dutifulness	0.055	0.005	-0.042	-0.036	0.084	-0.026	-0.035
AchievementStriving	0.064	0.099	-0.064	-0.253	-0.009	-0.015	-0.070
SelfDiscipline	0.096	0.127	0.084	-0.071	0.098	0.126	-0.015
Cautiousness	0.010	-0.015	-0.213	0.138	0.054	-0.082	-0.007
Neuroticism	-0.092	-0.101	0.033	-0.001	0.043	-0.008	0.076
Anxiety	-0.090	-0.122	0.069	0.104	-0.037	-0.209	0.059
Anger	-0.076	-0.011	0.011	0.147	0.092	-0.178	0.065
Depression	-0.083	-0.113	0.070	-0.091	-0.081	0.134	0.087
SelfConsciousness	-0.110	-0.196	-0.043	0.003	0.056	0.053	0.081
Immoderation	-0.055	-0.044	-0.036	-0.127	0.079	0.127	0.054
Vulnerability	-0.102	-0.050	0.047	-0.002	0.130	0.086	0.078
OpenessToExperience	0.012	-0.057	-0.208	0.008	0.023	-0.027	-0.050
Imagination	-0.036	-0.160	-0.159	0.024	0.205	0.029	0.032
Altruistic	0.064	-0.045	-0.107	-0.124	-0.009	-0.065	-0.078
Emotionality	0.022	-0.016	-0.059	0.042	-0.184	0.090	0.023
Adventurousness	0.067	-0.002	-0.021	-0.131	0.051	-0.068	-0.118
Intellect	0.001	0.002	-0.154	-0.097	0.031	-0.040	-0.117
Liberalism	-0.081	0.001	-0.174	0.155	-0.123	-0.093	0.052
HRV4	-0.051	0.113	-0.060	0.007	-0.125	-0.015	-0.015
HRV5	-0.078	0.084	0.011	-0.021	-0.042	0.022	-0.025
HRV6	-0.087	0.056	0.046	-0.118	-0.007	0.089	0.046

HRV7	-0.079	0.015	0.060	-0.070	0.034	0.122	0.087
HRV8	-0.070	0.005	0.068	-0.094	0.034	-0.033	0.115
HRV9	-0.071	-0.010	-0.024	-0.101	0.019	-0.162	0.112
HRV10	-0.067	0.001	-0.097	-0.002	0.004	-0.079	0.099
HRV11	-0.010	0.068	0.016	0.044	0.098	0.148	0.092
EMGQ1	-0.047	-0.071	0.127	-0.075	-0.004	0.021	0.005
EMGQ2	-0.104	-0.061	-0.029	-0.058	0.005	-0.039	-0.022
EMGQ3	-0.112	-0.047	0.068	0.030	0.045	0.016	-0.045
EMGQ4	-0.119	-0.060	0.042	-0.116	-0.038	-0.065	-0.009
EMGQ5	-0.111	-0.022	0.033	0.153	-0.056	-0.026	0.027
EMGQ6	-0.128	0.019	0.042	-0.039	0.007	0.026	-0.024
EMGQ7	-0.115	0.019	-0.053	-0.043	0.005	-0.058	0.022
EMGQ8	-0.118	-0.026	0.004	0.008	-0.031	0.135	0.011
EMGQ9	-0.122	-0.056	0.033	0.059	-0.002	0.010	0.025
EMGQ10	-0.132	-0.041	-0.025	0.025	-0.048	-0.071	0.058
EMGQ11	-0.145	-0.052	0.082	0.001	0.055	-0.150	-0.014
ENG1	-0.087	0.053	-0.054	-0.046	0.089	-0.048	-0.044
ENG2	-0.085	-0.057	0.021	0.085	0.114	0.026	-0.071
ENG3	-0.042	-0.076	-0.035	0.068	0.049	-0.187	-0.091
ENG4	-0.048	-0.064	0.081	0.136	0.200	0.044	-0.110
ENG5	-0.075	-0.079	-0.025	-0.119	0.037	0.018	-0.056
ENG6	-0.037	-0.092	-0.031	-0.049	0.073	0.029	-0.087
ENG7	-0.062	-0.026	-0.033	-0.015	0.106	-0.100	-0.052
ENG8	-0.059	-0.108	-0.029	-0.043	-0.084	0.004	-0.034
ENG9	-0.062	-0.105	-0.036	-0.089	-0.199	0.002	-0.054
ENG10	-0.043	-0.077	-0.035	0.031	-0.169	0.063	-0.040
ENG11	-0.064	-0.078	-0.044	-0.022	-0.065	0.042	-0.082
MT1	0.041	-0.046	0.180	0.004	-0.111	-0.030	-0.079
MT2	0.032	-0.018	0.041	0.065	-0.172	-0.061	-0.040
MT3	0.019	0.057	0.136	-0.035	0.082	-0.080	-0.079
MT4	0.021	0.046	0.063	-0.022	-0.012	-0.035	-0.071
MT5	0.012	0.056	0.029	0.044	-0.055	-0.164	-0.097
MT6	0.002	0.018	0.086	-0.012	-0.033	0.051	-0.096
MT7	0.014	0.020	-0.008	-0.015	-0.002	-0.124	-0.025
MT8	0.003	0.010	0.038	0.047	0.011	0.067	-0.062
MT9	-0.008	0.061	0.070	0.013	-0.017	0.195	-0.068
MT10	-0.019	0.002	0.031	-0.020	-0.009	0.112	-0.063

MT11	0.007	0.046	0.106	-0.020	0.036	0.040	-0.046
HR1	-0.089	0.076	0.005	0.035	-0.097	-0.151	**-0.196**
HR2	-0.107	0.035	-0.001	-0.068	-0.013	0.021	-0.178
HR3	-0.100	0.046	0.011	-0.040	0.007	-0.041	-0.183
HR4	-0.095	0.042	-0.021	0.016	-0.021	-0.014	**-0.190**
HR5	-0.110	0.014	-0.065	0.020	-0.038	0.022	-0.187
HR6	-0.101	0.001	-0.064	-0.010	-0.028	0.039	**-0.195**
HR7	-0.111	0.004	-0.068	0.016	-0.004	-0.032	-0.180
HR8	-0.114	-0.006	-0.073	0.031	-0.044	0.049	**-0.190**
HR9	-0.085	0.064	0.053	0.044	-0.051	0.043	-0.173
HR10	-0.061	0.077	0.109	0.027	-0.053	-0.040	**-0.192**
HR11	-0.114	0.003	-0.036	0.046	0.040	0.053	-0.171
Mu1	-0.022	-0.064	0.039	-0.011	-0.051	-0.016	0.017
Mu2	0.000	-0.078	0.064	0.035	0.065	-0.063	-0.032
Mu3	-0.006	-0.086	0.027	0.005	-0.061	-0.031	-0.008
Mu4	0.009	-0.079	0.040	0.034	0.033	-0.066	-0.017
Mu5	0.016	-0.071	0.041	-0.056	0.005	-0.115	0.004
Mu6	0.016	-0.057	0.080	0.063	0.025	-0.091	0.000
Mu7	0.014	-0.084	0.118	0.067	-0.003	-0.003	-0.032
Mu8	0.018	-0.083	0.079	-0.012	-0.010	-0.072	-0.026
Mu9	0.019	-0.098	0.072	-0.016	0.048	0.029	-0.030
Mu10	0.029	-0.070	0.083	0.043	-0.009	0.027	-0.030
Mu11	0.020	-0.085	0.042	0.033	-0.069	0.012	-0.028
PFG1	0.124	-0.030	0.099	0.134	0.049	0.011	-0.071
PFG2	0.126	-0.089	-0.083	-0.122	0.035	0.062	-0.027
PFG3	0.134	-0.124	-0.037	-0.117	0.061	0.022	-0.008
PFG4	0.153	-0.157	-0.082	-0.132	0.034	0.084	0.008
PFG5	0.151	-0.149	-0.021	0.041	-0.159	0.036	-0.043
PFG6	0.140	-0.113	-0.074	-0.068	-0.027	-0.004	-0.043
PFG7	0.152	-0.094	-0.112	-0.015	-0.133	-0.118	0.043
PFG8	0.164	-0.086	-0.046	0.012	-0.059	-0.008	0.036
PFG9	0.148	0.000	0.056	-0.119	0.025	-0.155	0.003
PFG10	0.138	-0.040	0.015	0.020	0.057	-0.045	0.035
PFG11	0.167	-0.022	-0.040	0.087	0.056	0.113	0.026
WL1	0.149	-0.040	0.032	0.095	-0.043	0.001	-0.066
WL2	0.124	-0.009	0.038	0.025	-0.040	-0.024	-0.119
WL3	0.133	-0.013	0.007	0.058	-0.034	0.126	-0.118

WL4	0.143	-0.005	0.009	0.047	0.023	-0.070	-0.086
WL5	0.135	0.010	-0.069	0.016	-0.017	-0.002	-0.097
WL6	0.143	-0.011	0.000	-0.061	-0.085	-0.060	-0.118
WL7	0.139	-0.011	0.017	-0.033	-0.026	-0.089	-0.059
WL8	0.148	-0.013	-0.060	0.001	-0.063	-0.045	-0.048
WL9	0.136	-0.007	0.009	-0.087	-0.024	-0.012	-0.092
WL10	0.131	-0.003	0.008	0.010	0.060	0.019	-0.102
WL11	0.128	-0.010	0.024	0.018	-0.005	0.033	-0.101
O1_AS1	-0.022	0.095	-0.067	0.058	-0.081	-0.015	0.085
O1_AS2	-0.014	0.097	-0.052	0.013	0.020	0.056	0.036
O1_AS3	-0.020	0.060	-0.081	-0.014	-0.054	0.064	0.040
O1_AS4	-0.017	0.104	-0.059	0.056	0.021	0.025	0.037
O1_AS5	-0.008	0.097	-0.081	0.008	0.023	-0.025	0.047
O1_AS6	-0.002	0.112	-0.062	0.010	0.009	0.036	0.048
O1_AS7	-0.011	0.072	-0.019	0.024	0.040	0.073	0.035
O1_AS8	-0.014	0.113	-0.087	-0.014	-0.058	-0.034	0.035
O1_AS9	-0.004	0.075	-0.084	-0.034	-0.023	0.066	0.033
O1_AS10	0.000	0.104	-0.054	0.026	-0.090	0.059	0.035
O1_AS11	0.001	0.096	-0.067	0.028	-0.049	-0.010	0.019
O2_AS1	-0.007	0.048	-0.046	-0.011	-0.030	-0.050	0.083
O2_AS2	-0.004	0.064	-0.009	-0.007	0.056	-0.038	0.016
O2_AS3	-0.003	0.030	-0.050	-0.016	0.014	0.001	0.025
O2_AS4	0.004	0.063	-0.025	0.009	0.052	-0.041	0.021
O2_AS5	0.014	0.056	-0.036	-0.047	0.102	-0.044	0.045
O2_AS6	0.022	0.077	-0.021	-0.026	0.003	-0.005	0.022
O2_AS7	-0.004	0.043	0.020	0.002	0.080	0.028	0.021
O2_AS8	0.004	0.062	-0.057	-0.061	-0.031	-0.094	0.026
O2_AS9	0.012	0.040	-0.038	-0.049	0.003	-0.008	0.014
O2_AS10	0.011	0.064	0.005	-0.010	-0.005	-0.063	0.015
O2_AS11	0.014	0.062	-0.031	-0.004	-0.028	-0.037	0.009
P3_AS1	-0.023	0.047	0.054	0.017	-0.046	-0.009	0.036
P3_AS2	-0.011	0.041	0.055	-0.020	0.047	0.012	-0.010
P3_AS3	-0.018	0.014	0.016	-0.061	-0.071	0.037	0.010
P3_AS4	-0.007	0.038	0.047	0.021	0.015	-0.042	-0.005
P3_AS5	0.001	0.034	0.008	-0.022	-0.005	-0.076	0.023
P3_AS6	0.006	0.046	0.027	0.002	-0.037	-0.021	0.016
P3_AS7	0.000	0.024	0.076	0.034	0.007	0.035	0.005

P3_AS8	0.001	0.050	0.001	-0.021	-0.104	-0.016	0.010
P3_AS9	0.010	0.015	0.033	-0.055	-0.020	0.076	-0.001
P3_AS10	0.017	0.046	0.052	0.014	-0.084	0.066	0.001
P3_AS11	0.012	0.034	0.032	0.001	-0.061	0.029	-0.010
P4_AS1	-0.015	-0.054	0.010	0.003	-0.019	-0.025	-0.009
P4_AS2	-0.007	-0.043	0.021	-0.016	0.098	-0.004	-0.046
P4_AS3	-0.009	-0.074	-0.015	-0.043	0.031	0.061	-0.034
P4_AS4	0.001	-0.051	0.005	-0.003	0.081	-0.009	-0.044
P4_AS5	0.010	-0.048	-0.022	-0.039	0.089	0.044	-0.014
P4_AS6	0.026	-0.035	0.016	-0.004	0.001	0.017	-0.033
P4_AS7	-0.005	-0.053	0.044	0.013	0.072	0.059	-0.031
P4_AS8	0.008	-0.059	-0.027	-0.058	-0.046	-0.051	-0.032
P4_AS9	0.017	-0.070	0.005	-0.042	0.019	0.086	-0.042
P4_AS10	0.020	-0.047	0.060	0.008	0.026	0.030	-0.041
P4_AS11	0.018	-0.048	0.003	0.017	-0.038	0.040	-0.048

Forward Step-wise Linear Multiple Regression Analyses

Narrative Reactions

PCA Component	Narrative Reactions: All Subjects R^2	Narrative Reactions: All Subjects Adjusted R^2	Narrative Reactions: Donated Only R^2	Narrative Reactions: Donated Only Adjusted R^2
1	0.08494628	0.064149605	0.118385075	0.059610746
2	0.308317065	0.276145766	0.354784929	0.262611348
3			0.716927513	0.651603093

Personality

PCA Component	Personality Traits: All Subjects R^2	Personality Traits: All Subjects Adjusted R^2	Personality Traits: Donated Only R^2	Personality Traits: Donated Only Adjusted R^2
1	0.066146438	0.042800099	0.207198199	0.146213445
2	0.391441917	0.36023381	0.491867438	0.407178678

Psychophysiology

PCA Component	Psychophysiology: All Subjects R^2	Psychophysiology: All Subjects Adjusted R^2	Psychophysiology: Donated Only R^2	Psychophysiology: Donated Only Adjusted R^2
1	0.035074068	0.014543729	0.402732869	0.36291506
2	0.122117687	0.083948891		
3	0.251015724	0.201083439		
4	0.286610144	0.221756521		
5	0.346058495	0.270018785		
6	0.359170841	0.267623818		
7	0.391315709	0.287394001		
8	0.416087918	0.299305502		
9	0.429255496	0.297545226		
10	0.549958035	0.43152594		
11	0.560483656	0.429816634		
12	0.577148471	0.436197961		

13	0.608261864	0.462759128
14	0.628135441	0.47501474
15	0.645208556	0.483939718
16	0.668162991	0.502244487
17	0.686841664	0.515109673
18	0.744266435	0.590826297
19	0.93027949	0.884600535

All Metrics Combined

	All Variables: All Subjects		All Variables: Donated Only	
PCA Component	R^2	Adjusted R^2	R^2	Adjusted R^2
1	0.114239314	0.091527502	0.396971017	0.350584172
2	0.178744168	0.135520177		
3	0.260569109	0.200615253		
4	0.3360962	0.262329111		
5	0.407275498	0.322600569		
6	0.495580096	0.406564819		
